# The Long-Term Health and Human Capital Consequences of Adverse Childhood Experiences in the Birth to Thirty Cohort: Single, Cumulative, and Clustered Adversity

**DOI:** 10.3390/ijerph19031799

**Published:** 2022-02-05

**Authors:** Sara N. Naicker, Marilyn N. Ahun, Sahba Besharati, Shane A. Norris, Massimiliano Orri, Linda M. Richter

**Affiliations:** 1 DSI-NRF Centre of Excellence in Human Development, University of the Witwatersrand, Johannesburg 2050, South Africa; shane.norris@wits.ac.za (S.A.N.); linda.richter@wits.ac.za (L.M.R.); 2 Department of Global Health and Population, Harvard T.H. Chan School of Public Health, Boston, MA 02115, USA; mahun@hsph.harvard.edu; 3 School of Public Health, Université de Montréal, Montreal, QC H3C 3J7, Canada; 4 Department of Psychology, School of Human and Community Development, University of the Witwatersrand, Johannesburg 2050, South Africa; sahba.besharati@wits.ac.za; 5 CIFAR Azrieli Global Scholars Program, CIFAR, Toronto, ON M5G 1M1, Canada; 6 SAMRC Developmental Pathways for Health Research Unit, University of the Witwatersrand, Johannesburg 2050, South Africa; 7 Hubert Department of Global Health, Rollins School of Public Health, Emory University, Atlanta, GA 30322, USA; 8 McGill Group for Suicide Studies, Department of Psychiatry, Douglas Mental Health University Institute, McGill University, Montreal, QC H3A 0G4, Canada; massimiliano.orri@mcgill.ca

**Keywords:** adverse childhood experiences, ACEs, human capital, birth cohort, clustered adversity

## Abstract

Human capital—that is the cumulative abilities, education, social skills, and mental and physical health one possesses—is increasingly recognized as key to the reduction of inequality in societies. Adverse childhood experiences have been linked to a range of human capital indicators, with the majority of research in high-income, western settings. This study aims to examine the link between adverse childhood experiences and adult human capital in a South African birth cohort and to test whether associations differ by measurement of adversity. Secondary analysis of data from the Birth to Thirty study was undertaken. Exposure data on adversity was collected prospectively throughout childhood and retrospectively at age 22. Human capital outcomes were collected at age 28. Adversity was measured as single adverse experiences, cumulative adversity, and clustered adversity. All three measurements of adversity were linked to poor human capital outcomes, with risk for poor human capital increasing with the accumulation of adversity. Adversity was clustered by quantity (low versus high) and type (household dysfunction versus abuse). Adversity in childhood was linked to a broad range of negative outcomes in young adulthood regardless of how it was measured. Nevertheless, issues of measurement are important to understand the risk mechanisms that underlie the association between adversity and poor human capital.

## 1. Introduction

Human capital is collectively the knowledge, skills, and health inputs accumulated across the lifespan that enables individuals to realize their full potential and contribute to the economic productivity of a society. It is manifest through a range of constructs, including educational attainment, physical and mental health, and social outcomes. It is well documented that human capital trajectories vary greatly within communities [1] and that one’s early experiences account for a substantial portion of the variation in adult human capital outcomes [2,3,4]. Another construct linked to economic productivity is adverse childhood experiences (ACEs), which is related to poorer health and social outcomes—or human capital—across the life course [5,6,7]. Early adversity in general, including child maltreatment, has long been established as detrimental for health and wellbeing [8,9]. Furthermore, the concept of ACEs—a quantifiable index of exposure to a range of adverse experiences, has in recent years been linked to a range of negative outcomes. The original ACE study found significant associations between ACEs and risk for alcoholism, drug abuse, smoking, risky sexual behavior, obesity, depression, suicide attempt, heart disease, cancer, chronic lung disease, liver disease, and skeletal fractures [10]. Following this seminal study, publications linking ACEs to one or more outcome grew exponentially [11] and systematic reviews and meta-analyses provided valuable overviews. One meta-analysis reviewed 37 studies and described links between greater exposure to ACEs and physical inactivity, overweight, obesity, diabetes, smoking, heavy alcohol use, sexual risk behavior, cancer, heart disease, respiratory disease, and mental ill health [7]. A second meta-analysis assessed 96 studies in which ACEs were examined against health and wellbeing outcomes. The study’s findings mirrored those of the previous meta-analysis, linking ACEs to a range of psychosocial, behavioral, and physical health outcomes [6]. Finally, a third meta-analysis on studies from Europe and North America not only connected ACEs to risk factors for ill health, but estimated the associated annual financial costs attributable to ACEs to be $581 billion in Europe and $748 billion in North America [12]. Identifying the specific ACEs or combinations of ACEs that are strongly linked to adult human capital outcomes may help elucidate the mechanisms of these associations and aid in developing targeted interventions to reduce the risk of poor human capital outcomes. 

However, the measurement of ACEs has important limitations. Studies typically rely on cumulative risk scores [13] or individual adversities measured through retrospective self-reports [14]. Evidence describes the relationship between ACEs and social and health problems as one that predicts the risk to increase in a strong and graded manner as the number and severity of ACEs increase [15,16]. Hence, a single adversity approach ignores the high probability that adversities co-occur and have an exponential impact. On the other hand, cumulative risk scores assume equal weighting of adversities while a number of studies have shown specific ACEs to be more deleterious than others [17,18,19]. Although there are currently no guidelines on the ACEs scoring in the available literature, some studies point to the ‘four or more’ cut-off functioning as a threshold level, with noticeable deviations in a range of outcomes at that mark [7]. As an alternative to both of these approaches, analyses of the patterning of ACEs recognize that the clustering and qualitative differences in combinations of ACEs are important for health and social outcomes and are linked to different consequences [20,21]. 

Another important limitation in ACEs research is the reliance on retrospective reports [6]. Previous research in longitudinal birth cohorts has demonstrated that prospective and retrospective reports of ACEs show poor agreement [22,23], similar to findings in this cohort [24], and are differentially linked to outcomes [17,23]. Meta-analysis findings conclude that prospective and retrospective measures of ACEs largely identify two different sets of individuals, cautioning that the measures should not be used interchangeably to study pathways of risk and outcomes. It is therefore important to compare findings based on prospective and retrospective measures in the same individuals.

Conceptually, frameworks such as the ACEs Pyramid—emanating from the ACEs study—attempt to explain the ways in which early adversity disrupts biological and psychological processes through interactions between genes and the environment [25]. The bio-developmental framework posits that early adversity precedes physiological maladaptations and disruptions due to either cumulative exposure or biological embedding during sensitive periods, leading to a range of poor health and wellbeing outcomes [26]. The ACEs Pyramid follows this logic but recognizes that exposure to ACEs is somewhat predetermined by social conditions and historical trauma in societies. ACEs then go on to disrupt neurodevelopment, giving rise to socio-emotional and cognitive impairments linked to the adoption of health risk behaviors that increase vulnerability to disease, disability, and social problems [10]. 

The objectives of this study are therefore to (a) examine the associations between ACEs and adult human capital, and (b) explore how the measurement of ACEs may vary in relation to these human capital outcomes. Prior research, including studies using this cohort, have highlighted sex differences in the prevalence of ACEs and their associations with outcomes [17,24,27,28,29,30]. The patterning of ACEs and their links to human capital outcomes will be disaggregated by sex throughout this analysis. To our knowledge, no study to date has investigated the relationship between ACEs and adult human capital outcomes, using these unique measurement methods, in low–middle income countries (LMICs).

## 2. Materials and Methods

### 2.1. Study Design and Participants

The Birth to Thirty study (Bt30, previously known as Birth to Twenty Plus) is a South African birth cohort of all singleton children born to mothers who were residents of Soweto, Johannesburg in a 7-week period of enrolment in 1990 [31]. The study is the largest and longest running birth cohort on the African continent, with an initial recruitment of 3273 participants, including their primary caregivers and subsequently a third generation born to the original cohort. The study has routinely followed participants through 23 data collection points over its 30-year lifespan, assessing growth, health, education, and wellbeing domains. At each of the data collection waves, well-trained field staff conducted face-to-face interviews with each participant for all interviewer-administered questionnaires and were available to assist with all self-administered questionnaires. A set of core questionnaires were routinely administered at each wave, assessing socio-demographics, household information, community and school environment, health and nutrition, risk behaviors, and more. The exposures and outcomes used in this study were pulled from selected sections in these questionnaires. Detailed descriptions of the cohort methods and sample have been published elsewhere [31,32,33,34]. For this study, data on 1436 participants included in the last data collection wave at age 28 were used. Ethics clearance was obtained from the Witwatersrand University Committee for Research on Human Subjects and written consent was obtained from all participants.

### 2.2. Exposures

ACEs in this study are defined as physical abuse, sexual abuse, emotional abuse or neglect, household dysfunction in the form of experience of divorce or parental separation, child separation, exposure to intimate partner violence (IPV), experience of living with a chronically ill or disabled individual or an individual with substance abuse problems, parental death, household legal trouble, and chronic household unemployment. ACEs were taken from a ‘life events’ section in the routine Bt30 questionnaire, which probed for recent major life events or changes (Supplementary Table S1). Retrospective reports of individual ACEs were created from a single report in the 22–23-year data collection wave. Prospective reports of individual ACEs were composed across the first 18 years of available data from caregiver reports (children aged 0–7 years old) and self-reported by the Bt30 participant thereafter.

### 2.3. Outcomes

Human capital outcomes were measured at age 28 and include both health and social measures conceptualized in a previous study [2].

#### 2.3.1. Education and Employment

Education refers to incomplete secondary schooling. Participants reported their highest school grade attained, dichotomized into complete (coded 0) or incomplete (1) secondary education. For the employment outcome, participations reported whether they were formally employed (i.e., had a work contract), (coded ‘0’) vs. not formally employed (‘1’). Both education and employment were single questions under their respective sub-sections in the routine Bt30 questionnaire at age 28.

#### 2.3.2. Welfare Receipt

Welfare receipt refers to a government cash transfer available to primary caregivers of children who qualify through an income means test. Welfare receipt in the form of a Child Support Grant (yes, coded ‘1’, vs. no, ‘0’) was recorded from administrative data supplied by the South African Social Security Agency. A single variable indicating whether or not the individual is receiving the grant was linked to the Bt30 data. Participants consented to the linking of this data through identity numbers. 

#### 2.3.3. Mental Health

Mental health was conceptualized as psychological distress and assessed using the World Health Organization’s Self Reporting Questionnaire. Items include 20 questions assessing symptoms experienced during the past month, such as “Do you feel nervous, tense or worried?” and “Do you sleep badly?”, coded in a binary manner and summed to obtain a total psychological distress score (alpha = 0.93). Participants in the top 20% of symptoms score were considered as having high psychological distress. 

#### 2.3.4. Social Isolation

Social isolation was assessed using 8 items based on the Inventory of Socially Supportive Behaviors, such as “How often you had someone who would listen to you when you needed to talk”, “Had someone you trust to talk with about your problems”. Items were answered on a 5-point scale from never to always, and then summed. Scores were dichotomized to identify participants who reported high levels of social isolation (scoring at the bottom decile of the distribution was coded ‘1’, versus those reporting lower levels, coded ‘0’). 

#### 2.3.5. Substance Abuse and Criminality

Substance abuse was derived from either reporting alcohol use more than 2–3 times a week and/or current use of non-medical drugs (including marijuana), scored as yes (‘1’) or no (‘0’). Criminality was assessed by asking participants whether, in the last year, they had been arrested, detained, jailed, or committed a crime without being caught, for example, stolen a car/motorbike, stolen in a shop or from a person, sold drugs or stolen goods, set property on fire or damaged/destroyed property, assaulted someone, or forced someone to have sex. A positive answer to any of the questions was coded ‘1’ versus ‘0’. These questions were taken from a section on ‘delinquency’ in the Bt30 routine questionnaire. 

#### 2.3.6. HIV Infection 

HIV status was assessed with a single question asking, “Have you ever tested positive for HIV?”, coded ‘1’ if yes and ‘0’ if no.

### 2.4. Covariates 

Covariates were included in this study based on their significance in the literature to multiple health and wellbeing outcomes [35,36], including in prior work on this cohort [2,17]. The covariates include sex; socio-economic status at participant birth, age 12, and age 22, measured as wealth quintiles derived from a list of assets (e.g., television, fridge, car, phone); maternal age at birth sorted into 4 age categories based on the distribution of maternal age (15–18, 19–24, 25–34, 35–46), and continuous measures of maternal and paternal years of schooling.

### 2.5. Statistical Analysis 

#### Measuring ACEs 

Single ACEs were included in the analyses as individual binary variables. Each ACE was coded ‘0’ for a negative response and ‘1’ for a positive response. 

To measure the effects of cumulative adversity, the individual binary ACEs were transformed into a categorical score with 5 levels for each participant, as follows: 0 = ‘no reported ACEs’, 1 = ‘one reported ACE’, 2 = ‘two reported ACEs’, 3 = ‘three reported ACEs’ and 4+ = ‘four or more reported ACEs’, following convention in the literature and allowing for comparability with other studies. The Bt30 sample has limited variability in its socio-economic status, similar low variability in the distribution of ACEs, and high prevalence of ACEs, given that the site of the study (i.e., Soweto, Johannesburg) is a previously socio-economically disadvantaged and low-income area. Most studies assessing either prospective or retrospective ACEs are based in high-income countries, often with populations with more heterogeneity in the distribution of ACEs [22]. To maximize within-cell counts and meaningfully analyze the data, cumulative ACEs were also categorized in a binary fashion as ‘0’ = ‘less than six’ and ‘1’ = ‘six or more’ reported ACEs. The ‘six or more ACEs’ cut-off was preferred over the conventional cut-off of ‘4 or more ACEs’ as this represents the mean ACE score for the Bt30 sample and takes into account the higher prevalence of ACEs in the sample. 

Clusters of ACEs were derived using latent class analysis (LCA), a mixture model technique that identifies groups of individuals (i.e., latent classes) on the bases of similarities in their pattern of co-occurrence of ACEs exposure. LCA was separately applied to the prospective and the retrospective reports. Several models were estimated with 2 to 6 latent classes and compared using the Bayesian Information Criteria (BIC, used as primary index), Akaike’s Information Criteria (AIC), and sample size-adjusted BIC (SSABIC). Lower values of the BIC, AIC, and SSABIC indicate a better fitting model. Entropy was also used to assess the distinction between classes, where values closer to 1 indicate good distinction (Supplementary Table S2). Once the best model was identified, participants were assigned to their most likely class, creating a categorical cluster variable. LCA was performed with Mplus version 8, with missing data on ACEs variables handled using Full Information Maximum Likelihood.

Associations between the 3 methods of measuring ACEs—single, cumulative, and clustered—and human capital outcomes—were examined using logistic regression. Two sets of models were fitted for each ACE measurement for both retrospective and prospective reports of ACEs: (i) a crude, unadjusted model (Supplementary Tables S3 and S4), followed by (ii) models adjusted for all covariates (Supplementary Tables S5 and S6). Sex was included as a covariate in all models but separate analysis by sex, excluding the variable at the covariate level, was conducted and is available in Supplementary Tables S7 and S8. To handle missing data on the covariates, we used multiple imputation by chained equations, so that models were estimated across 10 datasets and then pooled. All data management, multiple imputation, and regression analyses were conducted in Stata version 15.1. Data missingness for each of the ACEs, covariates, and outcomes is detailed in Table 1.

## 3. Results

### 3.1. Characteristics of the Sample 

Among the 1436 participants in the study, 47.5% were male and 52.4% were female (Table 1). Similar proportions of participants were born to young mothers (11.2% to mothers aged 15–18 at birth and 10.4% to mothers aged 35–44+ at birth), with the majority (78.3%) born to mothers aged between 19–34. Fathers had on average 1 year of additional schooling (mean = 10.5) than mothers (mean = 9.5). At birth, 15.4% of participants were in the lowest wealth quintile compared to 11.4% in the highest, at 12-years-old, 22.4% were in the lowest quintile and 19.0% in the highest, and at age 22, 31.4% fell into the lowest quintile and 13.9% in the highest, leaving a shrinking middle across quintile 2–4 from birth to age 22 of 73.1%, 58.5%, and 54.6%, respectively.

### 3.2. Prevalence of Human Capital Outcomes

The most prevalent adverse outcomes were unemployment (43.7%), incomplete secondary education (32.3%), substance use (26.9%), and welfare receipt (28.2%). Just over half of the females with children (51.4%) receive welfare compared to 1.3% of males caring for children. While both parents are eligible to receive welfare in the form of the Child Support Grant in South Africa, the greatest number of recipients (over 95% nationally and 98% in this sample), are women [37]. Females reported higher rates of psychological distress (23.7% compared to 10.9%), and HIV infection (16.8% compared to 11.1%) than males. Males reported higher rates of social isolation (13.7% compared to 7.5%), incomplete secondary education (39.1% compared to 25.3%), criminality (28.0% compared to 5.3%), and substance use (41.0% compared to 12.7%) compared to females.

### 3.3. Prevalence of ACEs

#### 3.3.1. Single ACEs

The most commonly reported prospective ACEs were chronic unemployment (86.2%), exposure to violence (70.4%), household death (63.1%), and household illness or disability (62.6%). Prospectively, 54.7% of participants reported physical abuse, 38.1% reported sexual abuse, and 35.7% reported emotional abuse. Reported exposure to all single ACEs, with the exception of parental death, decreased in retrospective reports. The most common retrospective ACEs were parental divorce (43.9%), chronic unemployment (42.6%), and household illness or disability (36.5%). Retrospectively, physical abuse was reported at a rate of 7.3%, sexual abuse at 3.8%, and emotional abuse or neglect at 34.8%.

#### 3.3.2. Cumulative ACEs

Similar patterns are seen in the prevalence of cumulative ACEs. While 87.0% of participants report four or more ACEs prospectively, 37.2% report four or more ACEs retrospectively. The proportion of participants reporting no ACEs remains low both prospectively (0.6%) and retrospectively (9.3%). Using the binary cut-off, 55.0% of participants report less than six ACEs prospectively, and 87.7% report less than six ACEs retrospectively.

#### 3.3.3. Clustered ACEs

The best fitting LCA models identified four classes for both the prospective and retrospective ACEs: low adversity (7.9% prospective, 41.8% retrospective); moderate adversity-dysfunction (39.9% prospective, 27.0% retrospective); moderate adversity-abuse (16.2% prospective, 16.3% retrospective); and high-adversity (36.0% prospective, 15.0% retrospective).

**Figure 1:**
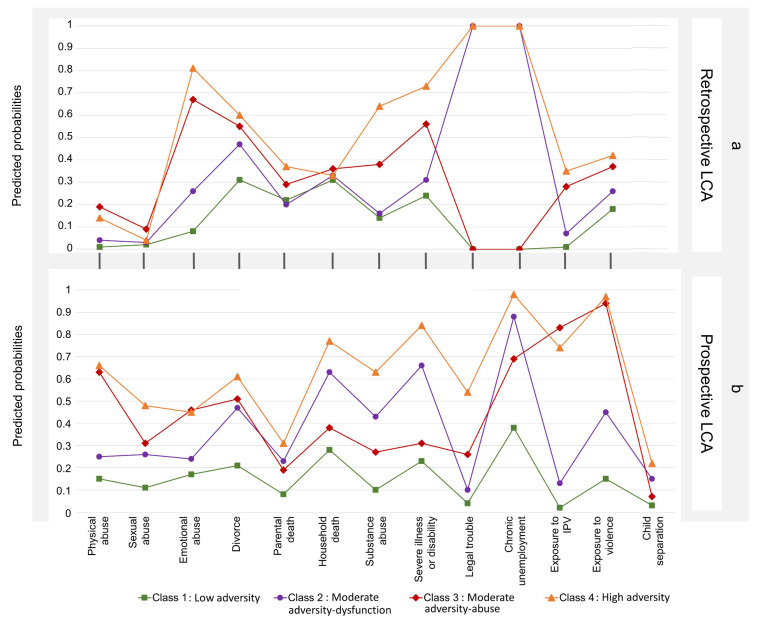
Predicted probabilities for latent class analyses of: (**a**) retrospective ACEs; (**b**) prospective ACEs.

Figure 1 shows the predicted probability of each adversity for each of the prospective and retrospective classes that led to the characterization of the classes. For prospective ACEs, *low adversity* was the smallest class, with the highest probabilities being a 28% chance of living in a household in which a member has died and 23% chance of living in a household where a member has a serious chronic illness or disability. Inclusion in the *moderate adversity-dysfunction* class was driven by ACEs related to household dysfunction, namely high chances of chronic unemployment (88%), parent divorce (47%), household death (63%), and household substance abuse (43%), among others. Participants in *moderate adversity-abuse* had a 66% chance of reporting physical abuse, 46% chance of reporting emotional abuse, as well as high levels of chronic unemployment (69% chance), exposure to IPV (83%), and community violence (94%). In the *high adversity* class, the probability of experiencing any one of the ACEs was greater than 40% for 11 of the 13 ACEs.

For retrospective clusters of ACEs, the *low adversity* group was characterized by highest probabilities for parental divorce and household death (31% each). Participants in the *moderate adversity-dysfunction* class had a 26% chance of reporting emotional abuse compared to participants in *moderate adversity-abuse* with a 66% chance of reporting emotional abuse. While those participants in *moderate adversity-abuse* were very unlikely to report chronic unemployment and household legal trouble compared to participants in *moderate adversity-dysfunction* who were almost certainly experiencing those two ACEs. The *high adversity* class had a more than a 40% chance of reporting seven of the 12 ACEs, with an 81% chance of emotional abuse, 73% chance of household illness/disability, 64% chance of household substance abuse, and 60% chance of parent divorce, and were more likely to report chronic unemployment (100%) and household legal trouble (100%).

### 3.4. Associations between ACEs and Human Capital Outcomes

**Figure 2:**
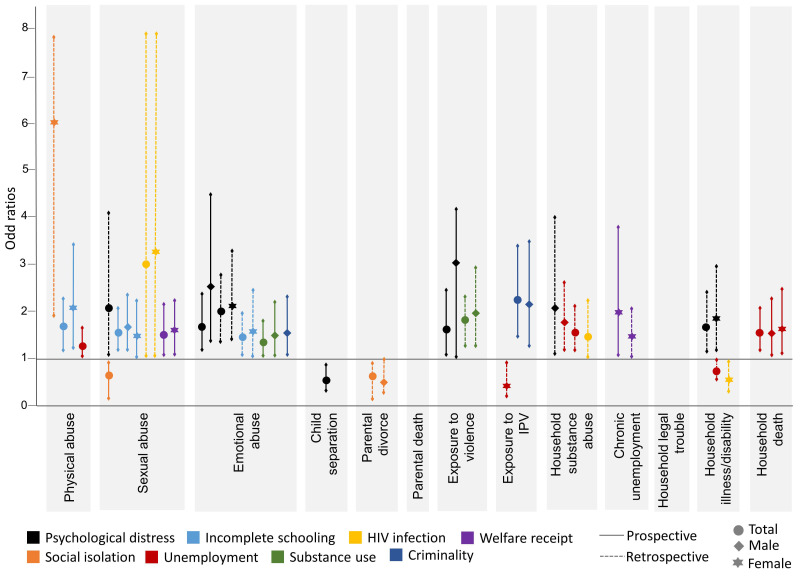
Significant adjusted associations (odds ratios) between single ACEs and human capital outcomes, for the total sample and disaggregated by sex.

**Figure 3:**
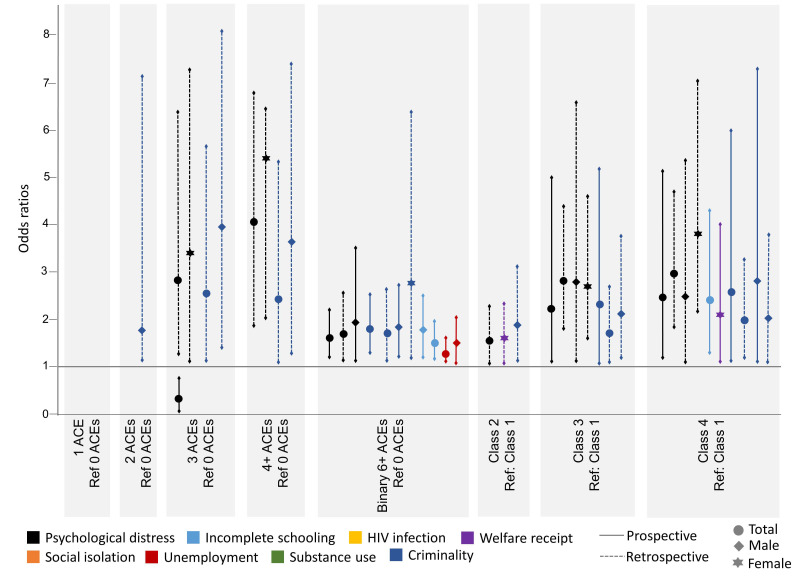
Significant adjusted associations (odds ratios) between cumulative and clustered ACEs and human capital outcomes, for the total sample and disaggregated by sex.

Figure 2 illustrates the significant findings from adjusted logistic regressions displayed for the associations between both prospective and retrospective single ACEs and human capital outcomes for the total sample, which are disaggregated by sex. Figure 3 shows the significant adjusted associations between prospective and retrospective cumulative and clustered ACEs and human capital outcomes, again for the total sample and disaggregated by sex.

#### 3.4.1. Single Adversities and Human Capital Outcomes

Prospective physical abuse was associated with greater likelihood of incomplete schooling (OR 1.69, CI 1.21–2.35) and unemployment (OR 1.31, CI 1.02–1.69) in the full sample. Females who reported prospective physical abuse had a greater likelihood of incomplete schooling (OR 2.08, CI 1.25–3.45) than their male counterparts (OR 1.69, CI 0.96–2.21) and females who reported retrospective physical abuse were 6.02 times (CI 1.93–8.85) more likely than males (OR 0.96, CI 0.33–2.74) to experience social isolation. Prospective sexual abuse was associated with welfare receipt (OR 1.53, CI 1.07–2.20) and incomplete schooling (OR 1.58, CI 1.21–2.08), and retrospective sexual abuse was associated with psychological distress (OR 2.05, CI 1.02–4.12) and HIV infection (OR 3.03, CI 1.03–8.91) in the full sample. Prospective emotional abuse was associated with greater odds of experiencing psychological distress (2.54 (CI 1.42–4.53) and substance use (1.52 (CI 1.01–2.25) for males only, while retrospective emotional abuse was associated with psychological distress (OR 2.15, CI 1.39–3.32) and incomplete schooling (OR 1.60, CI 1.02–2.49) for females only. Males were also 1.57 (CI 1.04–2.38) times more likely to report criminality if they prospectively reported emotional abuse. Prospective exposure to violence was associated with psychological distress (OR 1.64, CI 1.08–2.49), and again the association is stronger for males (OR 3.05, CI 1.01–9.18) than females (OR 1.52, CI 0.97–2.39). Retrospective exposure to violence was linked to substance abuse in males (OR 1.96, CI 1.30–2.96). Participants exposed to IPV in the home prospectively had 2.27 times (CI 1.49–3.45) greater likelihood of criminality and those who reported a death in the household were more likely to be unemployed (OR 1.57, CI 1.17–2.11). Males who reported substance abuse in the household retrospectively were 1.8 times (CI 1.21–2.66) more likely to be unemployed and 2.08 times (CI 1.09–3.99) more likely to experience psychological distress. Retrospective serious illness or disability in the household was associated with higher odds of psychological distress for females (OR 1.88, CI 1.18–2.98).

#### 3.4.2. Cumulative ACEs and Human Capital Outcomes

The risk for poorer outcomes increased along with the number of ACEs whether reported prospectively or retrospectively. Reporting more than six ACEs prospectively was significantly associated with greater odds of psychological distress (OR 1.63, CI 1.19–2.23), incomplete schooling (OR 1.51, CI 1.15–1.99), unemployment (OR 1.28, CI 1.10–1.64), and criminality (OR 1.80, CI 1.28–2.54) compared to reporting less than six ACEs in the full sample—all of which were only significant for males when disaggregated by sex (OR 1.97, CI 1.11–3.48; OR 1.74 CI 1.20–2.53; OR 1.47, CI 1.04–2.09; OR 1.84, CI 1.22–2.76, respectively). Reporting more than six ACEs retrospectively was associated with increased likelihood of psychological distress (OR 1.72, CI 1.13–1.32) and criminality (OR 1.69, CI 1.09–2.63) compared to less than six ACEs. Females retrospectively reporting three ACEs and four or more ACEs were 3.5 (CI 1.13–10.38) and 5.4 (CI 2.04–14.51) times more likely to report psychological distress, respectively. Females were 2.8 times (CI 1.18–6.5) more likely to engage in criminality if they experience more than six ACEs and males are more than three times more likely to engage in criminality when they report more than one ACE (OR 3.10, CI 1.05–9.14 for two ACEs; OR 3.97, CI 1.41–11.21 for three ACEs; and OR 3.64, CI 1.27–10.47 for more than four ACEs).

#### 3.4.3. Clustered ACEs and Human Capital Outcomes

Prospectively, compared to the low adversity cluster, the odds of experiencing psychological distress in the moderate adversity-dysfunction cluster are 2.8 (1.18–6.64) for males and 2.71 (1.58–4.63) for females. Individuals in the moderate adversity-abuse cluster are 2.23 times (1.10–5.04) more likely to experience psychological distress and 2.29 times (1.01–5.22) more likely to engage in criminality. Females who fall in the moderate adversity-abuse cluster have a greater likelihood of welfare receipt (OR 1.60, CI 1.06–2.41). The high adversity cluster has greater odds of experiencing psychological distress (OR 2.47, CI 1.19–5.09), incomplete schooling (OR 2.39, CI 1.33–4.32), and criminality (OR 2.60, CI 1.12–6.02).

Retrospectively, when comparing all other clusters to the low adversity group, there is a greater likelihood of psychological distress. The association is strongest in the high adversity cluster (OR 2.97, CI 1.86–4.74), followed by the moderate adversity-abuse group (OR 2.82, CI 1.8–4.41), and lastly the moderate adversity-dysfunction cluster (OR 1.52, CI 1.01–2.29). The moderate adversity-abuse group is also associated with criminality (OR 1.69, CI 1.04–2.73). Males in the high adversity cluster have increased odds for criminality (OR 2.84, CI 1.10–7.34) and females in the same group are more likely to be receiving welfare assistance (OR 2.11, CI 1.10–4.05).

## 4. Discussion

### 4.1. Adverse Childhood Experiences and Human Capital

This study aimed to examine the relationship between ACEs and adult human capital outcomes using unique and under-utilized measurement methods of ACEs in comparison to these human capital outcomes. To our knowledge, no other study has drawn on this methodological approach in studying ACEs and none were conducted in the LMIC context of sub-Saharan Africa. This study found that ACEs can be linked to poor human capital outcomes in young adulthood in an urban South African sample. There are a number of individual adversities that are independently associated with human capital outcomes, particularly the abuse-level variables. For example, incomplete schooling is associated with physical, sexual, and emotional abuse; social isolation is associated with physical abuse; and welfare receipt and HIV infection are associated with sexual abuse. The persistent and independent impact that these abuse-level experiences have on health and wellbeing is supported in previous analysis [17]. Individual household dysfunction adversities also play a role in poor human capital outcomes. Household substance abuse was linked to psychological distress and unemployment; exposure to violence was linked to psychological distress and substance use; and household death was linked to unemployment. The cumulative effects of adversity in childhood were also evident. Exposure to greater levels of ACEs, irrespective of their type, was linked to poorer outcomes. Similarly, the clustering of ACEs could be linked to negative human capital outcomes across low-high and dysfunction-abuse planes. There were stronger associations between negative outcomes and ACEs when the *high adversity* group was compared to the *low adversity* group, and similarly when the group likely to experience more abuse was compared to the group likely to experience more household dysfunction.

Globally, and particularly in LMICs such as South Africa, psychological distress contributes substantially to the burden of disease [38]. The prevalence of psychological distress—characterized here by depression, anxiety, and somatic symptoms—in this sample (17.3%) is lower than reported in a nationally representative South African survey (23.9%) [39], although the current study focused on participants expressing the highest levels of psychological distress. Experiences of sexual and emotional abuse, as well as exposure to violence, and substance abuse and severe illness/disability in the household as a child were risk factors for presenting with high psychological distress in adulthood. Individuals with more than three ACEs and in any cluster other than the *low adversity* group were significantly more likely to experience psychological distress. A growing body of research, including in this cohort [17], have linked ACEs to mental illness in various forms: internalizing and externalizing problems [40], depressive and anxiety disorders [41,42], and personality disorders [43,44]. Some of the possible mechanisms from early adversity to mental ill health include the disruption of adaptive emotion regulation processes, alterations in the structure and function of key areas of the brain, and the development of maladaptive coping strategies [45]. 

Social isolation, or the objective lack of interaction with others [46], was significantly associated with reported physical abuse in childhood in this study. The implications of prolonged social isolation on mental and physical health in younger populations is still emerging, but evidence of resultant cognitive decline in middle-aged populations [47] and mortality in mixed-aged populations [48,49] is available. As more and more research is conducted, driven by COVID-19-induced social isolation, the persistent and serious consequences of social isolation for health and wellbeing are being recognized [50].

The South African schooling system is divided into four phases, with mandatory attendance in the first three quarters and an optional 4^th^ phase that results in graduation out of the school system [51]. Despite high rates of enrollment in both primary and secondary schooling [52], the rates of incompletion for the latter are alarmingly high, with between 50–60% of learners not completing their secondary schooling [53]. Rates of secondary school incompletion in the current sample are much lower (32.2%) than the national average (~50.0%), but follow the same trend of females more likely to complete than males [54]. The capacity to progress out of school into tertiary education and/or employment is crucial for maximized human capital. Reports of physical abuse, sexual abuse, and emotional abuse in childhood as well as experiencing six or more ACEs cumulatively and being placed in the *high adversity* cluster are significant predictors for incomplete schooling in this study. Similar findings are seen in another birth cohort; retrospective physical and sexual abuse were significantly associated with failing to achieve secondary school qualifications, but these associations lost significance after adjusting for social, parental, and individual factors [55]. The pathway from abuse to educational attainment is partially through performance at school. Learners who reported abuse and maltreatment were likely to perform poorly on tests, repeat grades, and encounter more problems with schoolwork [56,57].

Inextricably linked to incomplete schooling is unemployment, given that income poverty and inequality in South Africa are driven by disparities in qualifications and skills [58]. South Africa has an average unemployment rate of 34.4%, with youth unemployment (15–24-year-olds) at 64.4% and young adult unemployment (25–34-year-olds) at 42.9% [59]. Young adults in this study have similar rates of unemployment at 43.7%, risk factors for which were physical abuse, household death, and household substance abuse, as well as reporting six or more ACEs. A cross-sectional study looking at retrospective ACEs found that adults with four or more ACEs were 2.3 times more likely to be unemployed compared to adults with no ACEs [60], and other studies have linked early adversity to unemployment in similar ways [61,62,63]. Associations between ACEs and human capital outcomes such as unemployment and incomplete schooling are difficult to tease out given the high youth unemployment rates and poorly ranked education system in South Africa [64]. There is likely an interplay between exposure to ACEs and contextual and structural factors. For example, with a national dependency ratio of 52.7% and 70% (59% in 2003) of children living in households with at least one working adult [65], a household death may impact household income, ability to attend school, and subsequent employment. In fact, one study estimates that a 1 percentage point increase in school attendance is associated with an average decrease of 6 percentage points in the dependency ratio [66].

Sexual abuse in childhood is independently associated with welfare receipt in adulthood for females, as was inclusion in the *moderate adversity-abuse* and *high adversity* groups. General social protection measures in South Africa have made substantial contributions to poverty reduction [67], and the Child Support Grant in particular has had multiple benefits for vulnerable children and families [68,69,70]. Nevertheless, the receipt of the grant is a good indication that the household is income- and resource-poor [71].

HIV infection was also independently associated with sexual abuse in childhood for females. Prevalence of HIV positive status in the sample was 14%, mirroring the national population at 14% and below the 15–49-year-old age group at 20% [72]. A cross-sectional study among 2042 post-natal women in Harare, Zimbabwe found that 15% of the women tested positive, and that women who reported child sexual abuse were three times as likely to test positive for HIV [73]. The mechanisms for infection could operate directly, with infection occurring as a result of forced sex or rape, or indirectly through the weakening of psychological wellbeing and other protective factors that could lead to risky behaviors [74,75]. A South African study found that physical, sexual, and emotional abuse and neglect were associated with a range of HIV-risk behaviors [76]. 

Substance use in adulthood was associated with emotional abuse and exposure to violence in childhood, but only for males in the sample. Substance abuse has long been a global health challenge contributing to personal disability and mortality and economic burdens to society [77]. Studies in South Africa and worldwide have linked childhood trauma directly and indirectly, through mediation, to substance use in adulthood [78,79]. Furthermore, a recent meta-analysis suggests that individuals with four or more ACEs were twice as likely to be current smokers or heavy drinkers and six times as likely to drink problematically than those with no ACEs [7]. Criminality was linked to emotional abuse and exposure to IPV in childhood, as was inclusion in the *moderate adversity-abuse* and *high adversity* clusters. Females were almost three times more likely to engage in criminality if they reported six or more ACEs whereas males were 3–4 times more likely with more than one ACE. A study on siblings demonstrated that the likelihood of committing a crime doubled with experiences of child abuse and neglect [80]. Compared to females, relatively low levels of adversity could be linked to criminality in males; however, our analyses did not differentiate between serious and petty, or violent and non-violent crime, which may contribute to these differences. Findings in the literature are divergent, with some studies arguing for similar propensities for females and males to engage in serious, persistent, and violent crime [81], a study where males were at greater risk of committing a violent offense [82], and another where females were more likely to be arrested for violence [83]. The co-occurrence of substance use and criminality is well documented [84,85], and recent research has examined pathways showing that for moderate-to-high substance users, ACEs are linked to increased criminality [86]. These findings are interesting and may partly explain the sex differences in expressions of criminality. 

### 4.2. Measuring ACEs 

Comparing different approaches to ACEs measurement reveals several insights with regard to human capital outcomes. Individual ACEs, particularly prospective physical, sexual, and emotional abuse and exposure to violence can be linked to poorer outcomes. Retrospectively, these single ACEs, as well as household dysfunction indicators, are associated with poorer outcomes. Part of this may be that children may not be fully aware of household dysfunction and its extent or severity, as it occurs prospectively, and they may piece together these reports in hindsight. Alternatively, only those participants who experienced severe household dysfunction may be reporting so retrospectively. Another possibility is that prospective and retrospective measures of ACEs may be identifying different groups of individuals within a sample. Consequently, those identified as having greater ACEs prospectively may have different pathways to poor outcomes than those identified as having had greater ACEs retrospectively [22]. 

Cumulative measures of ACEs show graded relationships with gradual increases in risk for poor outcomes, demonstrating their ability to show the snowballing effect of ACEs. Clustered ACEs improve on these measures through their ability to make qualitative distinctions between ACEs that tend to co-occur. However, the usefulness of the ACEs score as a rapid screening tool should not be ignored. Cut-offs for ACE categories should be made in consideration with the distribution of ACEs among the population. The conventional cut-off at four ACEs in the five-level ACEs categories does not appear to be adequate at distinguishing between those who are at a greater risk for poorer outcomes in high adversity settings. However, this five-level ACEs indicator, popularized by the CDC Kaiser study [10], appears to be more indicative of poorer outcomes when ACEs are assessed retrospectively compared to prospectively.

### 4.3. Implications of Findings 

Single ACEs at the individual level—physical, sexual, and emotional abuse—have persistent and long-term impacts on a range of human capital outcomes. Exposure to high levels of adversity accumulated over childhood can lead to equally poor outcomes in adulthood. Patterns of ACEs can differentially predict human capital outcomes—the two distinct patterns are low-high and dysfunction-abuse. Individuals who fall into a *high adversity* category, characterized by generalized adversity across a range of indicators, are prone to poorer human capital outcomes. Prospectively, individuals with a high likelihood of abuse and the co-occurrence of household dysfunction in the form of IPV, chronic unemployment, and exposure to violence, are linked to adverse mental health. Retrospectively, emotional abuse and some household dysfunction can be linked to poorer human capital outcomes even in the absence of poverty proxied by chronic unemployment. Individual ACEs and a moderate amount of adversity may contribute to resilience and protect against certain poor human capital outcomes. Both individual and cumulative ACEs—the same type and number of ACEs—appear to affect males and females in different ways, leading to different outcomes. Therefore, demonstrating that disaggregation by sex is important. In further support of this, one study that assessed gendered profiles of adversity concluded that there are separate and distinct patterns of childhood adversities, with females experiencing more complex and varied patterns [29].

### 4.4. Strengths and Limitations

The Bt30 sample is situated in a previously disadvantaged urban area in South Africa, limiting the generalizability of findings. The prevalence of ACEs, both prospectively and retrospectively, is considerably higher than global and meta-analytic estimates [7]. However, ACEs evidence in low-income, high-violence settings with widespread adversity across the life course is slowly emerging [19,87,88,89] and highlighting important similarities and differences in the field. Bt30 is one of few cohorts in an LMIC that has reached young adulthood—the period in which human capital disparities are likely to emerge [90]. This cohort is also one of few with both prospective and retrospective data on ACEs, especially in LMIC contexts; comparisons between prospective and retrospective reports of ACEs are key to understanding the risk mechanisms that underlie poor outcomes. This is particularly true given limitations around self-reported retrospective data which can be open to social desirability bias, recall error and the like. The authors concede that the retrospective self-reports of ACEs may be exposed to such bias but counter that (a) self-reports may be closer to true estimates, taking into account unreported and unobserved adversity [91], and (b) that both self-reported retrospective and prospective measures of adversity show substantial links to poor outcomes [92], supporting the usefulness of these self-reported retrospective accounts of adversity.

## 5. Conclusions

The measurement of ACEs is more complicated than often assumed. Both timing—prospective and retrospective reports—and the approaches of measuring ACEs can give differing insights into their links to adverse outcomes. Consequently, thought needs to be given to how ACEs are used in practice and policy.

Given South Africa’s strained economy—similar to other LMICs—it is critical that efforts are made to cultivate and protect human capital. The prevention of abuse in childhood—physical, sexual, and emotional abuse—must be a priority. Families need to be supported to mitigate the effects of household dysfunction. Evans and Kim suggest that “cumulative rather than singular exposure to a confluence of psychosocial and physical environmental risk factors is a potentially critical aspect of the environment of childhood poverty” (p. 77) [93]. For resource-poor countries, understanding the potential impact of early adversity across the life course is critical to breaking the intergenerational cycle of poverty. 

## Figures and Tables

**Table 1:** Description of the study sample (*n* = 1436).

Variable	Male	Female	Total	Missing
**Exposures**				
**Single Prospective ACEs**				
Physical abuse	400 (58.57)	381 (50.73)	781 (54.65)	2 (0.14)
Sexual abuse	267 (39.09)	278 (37.17)	545 (38.13)	5 (0.35)
Emotional abuse	245 (35.98)	265 (35.43)	510 (35.71)	7 (0.49)
Child separation	92 (14.00)	120 (16.28)	212 (15.14)	42 (2.92)
Parental divorce	332 (49.63)	392 (52.55)	724 (51.09)	21 (1.46)
Parental death	160 (23.74)	152 (20.27)	312 (22.01)	12 (0.84)
Household death	416 (64.63)	463 (61.49)	879 (63.06)	8 (0.56)
Household substance abuse	305 (46.42)	355 (48.30)	660 (47.36)	44 (3.06)
Household illness/disability	413 (62.86)	460 (62.42)	873 (62.64)	42 (2.92)
Household legal trouble	292 (43.20)	228 (30.32)	520 (36.76)	8 (0.56)
Chronic unemployment	567 (86.30)	634 (86.02)	1201 (86.16)	42 (2.92)
Exposure to IPV	372 (54.55)	285 (37.95)	657 (46.25)	3 (0.21)
Exposure to violence	530 (77.71)	474 (63.03)	1004 (70.37)	2 (0.14)
**Prospective ACE category**				0 (0.0)
0 ACEs	3 (0.44)	5 (0.66)	8 (0.55)	
1 ACE	12 (1.76)	14 (1.86)	26 (1.81)	
2 ACEs	13 (1.90)	30 (3.98)	43 (2.94)	
3 ACEs	49 (7.17)	62 (8.23)	111 (7.70)	
4+ ACEs	606 (88.73)	642 (85.26)	1248 (86.99)	
**Prospective Binary ACE score**				0 (0.0)
Less than 6 ACEs	348 (50.95)	442 (58.70)	790 (55.01)	
6 or more ACEs	335 (49.05)	311 (42.30)	64,699 (44.99)	
**Prospective LCA derived ACEs**				0 (0.0)
Class 1: Low adversity	43 (6.30)	70 (9.30)	113 (7.87)	
Class 2: Moderate adversity-dysfunction	223 (32.65)	350 (46.48)	573 (39.90)	
Class 3: Moderate adversity-abuse	127 (18.59)	106 (14.08)	233 (16.23)	
Class 4: High adversity	290 (42.46)	227 (30.15)	517 (36.00)	
**Single Retrospective ACEs**				
Physical abuse	58 (8.90)	41 (5.75)	99 (7.33)	71 (4.94)
Sexual abuse	16 (2.47)	36 (5.05)	52 (3.76)	74 (5.15)
Emotional abuse	251 (37.19)	241 (32.44)	492 (34.82)	18 (1.25)
Parental divorce	251 (43.35)	276 (44.44)	527 (43.90)	236 (16.43)
Parental death	184 (27.34)	167 (22.57)	351 (24.96)	23 (1.60)
Household death	191 (33.39)	199 (32.20)	390 (32.80)	246 (17.13)
Household substance abuse	205 (30.37)	178 (23.96)	383 (27.17)	18 (1.25)
Household illness/disability	240 (35.50)	277 (37.48)	517 (36.49)	21 (1.46)
Household legal trouble	172 (25.48)	144 (19.38)	316 (22.43)	18 (1.25)
Chronic unemployment	306 (45.33)	296 (39.84)	602 (42.59)	18 (1.25)
Exposure to IPV	81 (12.05)	97 (13.18)	178 (12.62)	28 (1.95)
Exposure to violence	222 (33.04)	160 (21.83)	382 (27.44)	31 (2.16)
**Retrospective ACE category**				
0 ACEs	58 (8.49)	76 (10.09)	134 (9.29)	
1 ACE	117 (17.13)	135 (17.93)	252 (17.53)	
2 ACEs	98 (14.35)	168 (22.31)	266 (18.33)	
3 ACEs	126 (18.45)	127 (16.87)	253 (17.66)	
4+ ACEs	284 (41.58)	247 (32.80)	531 (37.19)	
**Retrospective Binary ACE score**				0 (0.0)
Less than 6 ACEs	582 (85.21)	677 (89.91)	1259 (87.67)	
6 or more ACEs	101 (14.79)	76 (10.09)	177 (12.33)	
**Retrospective LCA derived ACES**				0 (0.0)
Class 1: Low adversity	268 (39.24)	332 (44.09)	600 (41.78)	
Class 2: Moderate adversity-dysfunction	184 (26.94)	203 (16.96	387 (26.95)	
Class 3: Moderate adversity-abuse	109 (15.96)	125 (16.60)	234 (16.30)	
Class 4: High adversity	122 (17.86)	93 (12.35)	215 (14.97)	
**Outcomes**				
Psychological distress	58 (10.86)	143 (23.68)	201 (17.27)	298 (20.75)
Social isolation	73 (13.67)	45 (7.45)	118 (10.56)	298 (20.75)
Incomplete secondary education	207 (39.06)	152 (25.33)	359 (32.20)	306 (21.31)
Unemployed	242 (45.32)	254 (42.12)	496 (43.72)	299 (20.82)
Welfare receipt ^1^	5 (1.28)	246 (51.36)	251 (28.82)	565 (39.35)
Criminality	150 (28.04)	32 (5.30)	182 (16.67)	297 (20.68)
Substance use	210 (41.02)	73 (12.74)	283 (26.88)	351 (24.44)
HIV infection	52 (11.13)	96 (16.78)	148 (13.96)	397 (27.65)
**Covariates**				
Sex	683 (47.56)	753 (52.44)	1436 (100.00)	0 (0.0)
Socio-economic status at birth				115 (8.01)
Quintile 1	101 (16.19)	103 (14.78)	204 (15.49)	
Quintile 2	112 (17.95)	126 (18.08)	238 (18.02)	
Quintile 3	214 (34.29)	240 (34.43)	454 (34.36)	
Quintile 4	135 (21.63)	139 (19.94)	274 (20.79)	
Quintile 5	62 (9.94)	89 (12.77)	151 (11.36)	
Socio-economic status at 12 years				380 (26.46)
Quintile 1	114 (23.12)	123 (21.85)	237 (22.49)	
Quintile 2	164 (33.27)	169 (30.02)	333 (31.65)	
Quintile 3	72 (14.60)	88 (15.63)	160 (15.12)	
Quintile 4	54 (10.95)	71 (12.61)	125 (11.78)	
Quintile 5	89 (18.05)	112 (19.89)	201 (18.97)	
Socio-economic status at 22 years				39 (2.72)
Quintile 1	209 (31.48)	230 (31.38)	439 (31.43)	
Quintile 2	106 (15.96)	116 (15.83)	222 (15.90)	
Quintile 3	154 (23.19)	139 (18.96)	293 (21.08)	
Quintile 4	109 (16.42)	139 (18.96)	248 (17.69)	
Quintile 5	86 (12.95)	109 (14.87)	195 (13.91)	
Maternal age at birth of child				2 (0.14)
15–18 years	72 (10.54)	89 (11.85)	161 (11.20)	
19–24 years	233 (34.11)	254 (33.82)	487 (33.97)	
25–34 years	305 (44.66)	331 (44.07)	636 (44.37)	
35–46 years	73 (10.69)	77 (10.25)	150 (10.47)	
Maternal education, mean (SD)	9.57 (2.63)	9.71 (2.60)	9.64 (2.62)	106 (7.38)
Paternal education, mean (SD)	10.49 (2.43)	10.62 (2.47)	10.56 (2.43)	426 (29.67)

^1^ Welfare receipt calculated for the sub-sample who have children (*n* = 871).

## Data Availability

Bt30 is housed in the DSI-NRF Centre of Excellence in Human Development at the University of the Witwatersrand and requests for data can be made through https://www.wits.ac.za/coe-human/open-access-datasets/ (accessed on 15 January 2022). Alternatively, the corresponding author of the study can be contacted for access to the data.

## Supplementary Materials

The following supporting information can be downloaded at: https://www.mdpi.com/article/10.3390/ijerph19031799/s1, Table S1: Adverse childhood experiences questionnaire; Table S2: Model fit indices for latent class analysis of prospective and retrospective ACEs; Table S3: Crude associations between single, cumulative and clustered prospective ACEs and health and human capital outcomes; Table S4: Crude associations between single, cumulative and clustered retrospective ACEs and health and human capital outcomes; Table S5: Adjusted associations between single, cumulative and clustered prospective ACEs and health and human capital outcomes; Table S6: Adjusted associations between single, cumulative and clustered retrospective ACEs and health and human capital outcomes; Table S7: Adjusted associations between single, cumulative and clustered prospective ACEs and health and human capital outcomes, by sex; Table S8: Adjusted associations between single, cumulative and clustered retrospective ACEs and health and human capital outcomes, by sex.

## Abbreviations

ACEs	Adverse Childhood Experiences
AIC	Akaike’s Information Criteria
BIC	Baysian Information Criteria
Bt30	Birth to Thirty
CI	Confidence intervals
DSI-NRF	Department of Science and Innovation-National Research Foundation
HIV	Human immunodeficiency virus
IPV	Intimate partner violence
LCA	Latent class analysis
LMIC	Lower-middle-income country
OR	Odds ratios
SSABIC	Sample-sex adjusted Bayesian Information Criteria
