# Supplementary material for: The Long-Term Health and Human Capital Consequences of Adverse Childhood Experiences in the Birth to Thirty Cohort: Single, Cumulative, and Clustered Adversity
Source: Int J Environ Res Public Health. 2022 Feb 5;19(3):1799. doi: 10.3390/ijerph19031799 (PMC8835353; doi:10.3390/ijerph19031799)
Supplement: Supplementary file 1 [file ijerph-19-01799-s001.zip › ijerph-1568373-supplementary.pdf]

Supplementary Material

Supplementary Table S1: Adverse childhood experiences questionnaire

**Adverse childhood experiences: Thinking back to your childhood, the first 18 years of your life, please tick each one that applies to you**			
ACE	**Question**	**Yes**	**No**
**Physical abuse**	Did a parent or other adult in the household **often or very often**…		
	Push, grab, slap, or throw something at you?		
	**OR**		
	**Ever** hit you so hard that you had marks or were injured?		
**Sexual abuse**	Did an adult or person at least 5 years older than you **ever**…		
	Touch or fondle you or have you touch their body in a sexual way?		
	**OR**		
	Attempt or actually have oral, anal, or vaginal intercourse with you?		
**Emotional abuse**	Did a parent or other adult in the household **often or very often**…		
	Swear at you, insult you, put you down, or humiliate you?		
	**OR**		
	Act in a way that made you afraid that you might be physically hurt?		
**Emotional neglect**	Did you **often or very often** feel that …		
	No one in your family loved you or thought you were important or special?		
	**OR**		
	Your family didn't look out for each other, feel close to each other, or support each other?		
**Physical neglect**	Did you **often or very often** feel that …		
	You didn't have enough to eat, had to wear dirty clothes, and had no one to protect you?		
	**OR**		
	Your parents were too drunk or high to take care of you or take you to the doctor if you needed it?		
**Parental divorce**	Were your parents **ever** separated or divorced?		
**Witnessing domestic violence**	Was your mother or stepmother:		
	**Often or very often** pushed, grabbed, slapped, or had something thrown at her?		

Supplementary Table S1: Adverse childhood experiences questionnaire

**Adverse childhood experiences: Thinking back to your childhood, the first 18 years of your life, please tick each one that applies to you**	
	**OR**
	**Sometimes, often, or very often** kicked, bitten, hit with a fist, or hit with something hard?
	**OR**
	**Ever** repeatedly hit at least a few minutes or threatened with a gun or knife?
**Alcohol and/or drug abuse in the household**	Did you live with anyone who was a problem drinker or alcoholic or who used street drugs?
**Mental illness in the household**	Was a household member depressed or mentally ill, or did a household member attempt suicide?
**Legal trouble in the household**	Did a household member go to prison?
**Chronic illness (other than mental illness) in the household**	Was there anyone in your household who was chronically ill when you were a child?
**Unemployment of parent/caregiver**	Was one or more of your parents/caregivers mostly unemployed during your childhood because they could not get a job?
**Death of parent/caregiver**	Did either of your parents/caregivers pass away before you turned 18?

Supplementary Table S2: Model fit indices for latent class analysis of prospective and retrospective ACEs

**Number of classes**	**AIC**	**BIC**	**SSABIC**	**Entrophy**	**Smallest class**
**Prospective LCA**					
Class 1	37452,01	37529,97	37488,66		
Class 2	35800,92	35962,84	35877,05	0,557	49,2
Class 3	35565,64	35811,51	35681,24	0,495	31,6
Class 4	35412,33	35742,17	35567,41	0,499	11,2
Class 5	35372,72	35786,51	35567,27	0,464	10,2
Class 6	35354,67	35852,42	35588,70	0,493	4,3
**Retrospective LCA**					
Class 1	18434,05	18499,91	18461,79		
Class 2	16348,06	16485,28	16405,86	0,797	33,7
Class 3	16156,52	16365,09	16244,37	0,688	13,0
Class 4	16018,37	16298,30	16136,28	0,632	12,0
Class 5	16013,32	16364,61	16161,29	0,663	1,8
Class 6	16015,12	16437,76	16193,14	0,647	1,8

Supplementary Table S3: Crude associations between single, cumulative and clustered prospective ACEs and health and human capital outcomes

	Psychological distress	Social isolation	Incomplete schooling	Unemployed	Substance use	HIV infection	Welfare receipt	Criminality
**Single ACEs**								
Physical abuse	1.18 [.85-1.64]	1.20 [.80-1.79]	1.73 [1.28-2.33]***	1.38 [1.08-1.76]**	1.03 [.76-1.40]	1.31 [.86-1.98]	1.28 [.92-1.78]	.88 [.60-1.27]
Sexual abuse	1.01 [.72-1.40]	.71 [.47-1.09]	1.74 [1.35-2.24]***	1.36 [1.05-1.77]*	.84 [.62-1.14]	1.47 [1.03-2.10]*	1.56 [1.16-2.10]**	1.08 [.76-1.53]
Emotional abuse	1.70 [1.23-2.35]**	1.16 [.79-1.71]	1.08 [.83-1.40]	1.04 [.80-1.33]	1.31 [.99-1.71]	1.25 [.87-1.81]	.86 [.62-1.18]	1.38 [1.00-1.91]*
Child separation	.65 [.42-1.02]	1.10 [.62-1.94]	.97 [.68-1.37]	1.06 [.76-1.48]	1.25 [.88-1.79]	1.14 [.69-1.87]	1.25 [.84-1.85]	1.09 [.68-1.74]
Parental divorce	.86 [.63-1.17]	.91 [.62-1.35]	1.15 [.88-1.51]	1.21 [.94-1.55]	.98 [.73-1.30]	1.08 [.76-1.52]	1.43 [1.04-1.97]*	.73 [.53-1.01]
Parental death	1.02 [.67-1.55]	.94 [.47-1.54]	1.25 [.94-1.66]	.89 [.66-1.20]	.94 [.66-1.34]	1.29 [.88-1.89]	.87 [.60-1.27]	1.26 [.86-1.87]
Exposure to violence	1.47 [.99-2.18]	.99 [.59-1.67]	.90 [.65-1.24]	.98 [.73-1.33]	1.47 [1.00-2.17]*	.80 [.51-1.27]	.81 [.58-1.13]	1.64 [1.04-2.58]*
Exposure to IPV	1.05 [.74-1.50]	1.23 [.81-1.86]	1.19 [.89-1.58]	.95 [.72-1.33]	1.52 [1.10-2.10]*	1.19 [.79-1.78]	.77 [.54-1.10]	2.48 [1.70-3.61]***
Household substance abuse	1.29 [.93-1.79]	1.19 [.81-1.86]	1.04 [.81-1.33]	1.07 [.84-1.35]	.81 [.59-1.10]	.88 [.61-1.25]	1.10 [.81-1.49]	.77 [.54-1.09]
Chronic unemployment	.85 [.54-1.34]	1.38 [.72-2.67]	1.29 [.83-1.99]	1.22 [.85-1.76]	.77 [.50-1.19]	.73 [.41-1.29]	1.79 [1.06-3.01]*	.70 [.43-1.13]
Household legal trouble	1.22 [.87-1.71]	1.53 [.99-2.36]	1.27 [.95-1.68]	1.01 [.79-1.29]	1.23 [.89-1.70]	.95 [.64-1.43]	.71 [.49-1.02]	1.56 [1.08-2.26]*
Household illness/disability	1.06 [.76-1.48]	.75 [.50-1.14]	1.20 [.91-1.58]	1.01 [.77-1.32]	.86 [.63-1.17]	1.13 [.77-1.65]	1.26 [.88-1.82]	1.13 [.79-1.63]
Household death	1.15 [.83-1.60]	.87 [.59-1.27]	.79 [.60-1.04]	1.43 [1.09-1.88]**	1.32 [.95-1.83]	1.10 [.76-1.58]	.90 [.62-1.32]	1.14 [.82-1.58]
**Cumulative ACEs**								
Less than 6	Ref	Ref	Ref	Ref	Ref	Ref	Ref	Ref
6 or more	1.61 [1.20-2.16]**	1.19 [.83-1.72]	1.67 [1.31-2.12]***	1.40 [1.10-1.77]**	1.39 [1.03-1.89]*	1.36 [.97-1.89]	1.12 [.86-1.45]	1.79 [1.32-2.42]***
0	Ref	Ref	Ref	Ref	Ref	Ref	Ref	Ref
1	.46 [.10-2.13]	N/A	1.61 [.15-16.87]	1.28 [.20-8.20]	1.41 [.13-15.75]	N/A	1.29 [.11-15.61]	.48 [.03-8.26]
2	.74 [.27-1.90]	.23 [.01-4.20]	2.11 [.22-19.75]	1.74 [.30-9.99]	2.26 [.24-21.44]	.79 [.12-4.69]	1.57 [.15-16.09]	1.12 [.11-11.25]
3	.42 [.19-.91]*	.93 [.10-8.40]	2.12 [.25-18.34]	1.98 [.38-10.34]	1.57 [.18-14.05]	.40 [.07-2.31]	1.71 [.19-15.75]	.88 [.10-7.81]
4+	N/A	.83 [.10-6.83]	3.52 [.43-28.72]	2.35 [.47-11.67]	2.39 [.28-20.05]	.51 [.10-2.53]	2.09 [.24-17.83]	1.43 [.17-11.71]
**Clustered ACEs**								
Class 1	Ref	Ref	Ref	Ref	Ref	Ref	Ref	Ref
Class 2	1.41 [.68-2.94]	1.15 [.50-2.65]	1.83 [1.03-3.24]*	1.45 .88-2.38]	.94 [.56-1.58]	1.05 [.49-2.25]	1.65 [.90-3.02]	1.16 [.52-2.57]
Class 3	1.94 [.88-4.24]	1.64 [.68-4.01]	1.83 [1.02-3.27]*	1.29 [.76-2.19]	1.60 [.89-2.86]	1.27 [.56-2.84]	1.11 [.55-2.23]	2.59 [1.19-5.65]*
Class 4	2.23 [1.13-4.38]*	1.55 [.69-3.45]	2.97 [1.70-5.18]***	1.90 [1.16-3.13]*	1.66 [.95-2.92]	1.37 [.66-2.86]	1.61 [.84-3.07]	2.77 [1.29-5.95]**

*$p<.05$ **$p<.01$ ***$p<.001$

N/A = omitted due to within cell sizes

The table shows the fold-increase in the odds (OR, odds ratio) of each adult human capital outcome (columns) for each level of ACEs measurement (rows). Models were run separately for each of the 3 ACE measurements

Supplementary Table S4: Crude associations between single, cumulative and clustered retrospective ACEs and health and human capital outcomes

	Psychological distress	Social isolation	Incomplete schooling	Unemployed	Substance use	HIV infection	Welfare receipt	Criminality
**Single ACEs**								
Physical abuse	1.06 [.63-1.80]	2.12 [1.06-4.22]*	1.10 [.64-1.88]	.98 [.62-1.57]	1.93 [1.11-3.35]*	.97 [.50-1.88]	.60 [.31-1.15]	1.58 [.87-2.86]
Sexual abuse	2.30 [1.21-4.39]*	1.58 [.68-3.69]	1.05 [.51-2.17]	.89 [.45-1.78]	1.25 [.60-2.60]	2.84 [1.04-7.75]*	1.50 [.65-3.47]	1.33 [.61-2.89]
Emotional abuse	1.90 [1.35-2.68]***	.83 [.68-3.69]	1.57 [1.19-2.08]**	1.09 [.84-1.41]	1.00 [.70-1.43]	1.32 [.82-2.11]	1.09 [.80-1.50]	1.32 [.95-1.84]
Parental divorce	.79 [.58-1.08]	.63 [.41-.96]*	.83 [.64-1.06]	1.06 [.78-1.45]	.92 [.66-1.30]	.87 [.52-1.47]	1.17 [.80-1.71]	.91 [.62-1.32]
Parental death	1.09 [.75-1.57]	1.37 [.85-2.22]	1.28 [.95-1.74]	.96 [.70-1.30]	.81 [.56-1.18]	1.11 [.76-1.61]	.78 [.53-1.15]	1.39 [.96-1.99]
Exposure to violence	.93 [.64-1.34]	1.41 [.90-2.21]	.99 [.75-1.32]	.96 [.72-1.27]	1.97 [1.47-2.64]***	.90 [.57-1.42]	.75 [.54-1.02]	1.71 [1.15-2.52]**
Exposure to IPV	.99 [.62-1.56]	.54 [.27-1.08]	.83 [.56-1.21]	.65 [.44-.97]*	1.02 [.64-1.64]	.69 [.38-1.24]	.88 [.56-1.38]	1.14 [.71-1.81]
Household substance abuse	1.30 [.92-1.83]	1.81 [1.18-2.76]**	1.22 [.87-1.72]	1.73 [1.33-2.25]***	1.14 [.81-1.59]	1.63 [1.12-2.38]*	.91 [.62-1.34]	1.15 [.80-1.65]
Chronic unemployment	1.10 [.79-1.53]	1.36 [.91-2.05]	1.21 [.91-1.59]	1.31 [1.03-1.66]*	.98 [.74-1.29]	.81 [.54-1.20]	1.31 [.99-1.74]	1.29 [.94-1.76]
Household legal trouble	.91 [.62-1.33]	.73 [.44-1.20]	1.00 [.74-1.36]	.96 [.68-1.35]	1.20 [.81-1.78]	1.22 [.80-1.86]	.90 [.61-1.34]	1.13 [.79-1.63]
Household illness/disability	1.75 [1.23-2.48]**	.79 [.51-1.22]	.91 [.68-1.21]	.76 [.58-.98]*	.72 [.54-.96]	.73 [.49-1.09]	1.08 [.77-1.51]	.85 [.54-1.34]
Household death	.95 [.67-1.33]	1.04 [.70-1.56]	.89 [.67-1.18]	1.16 [.87-1.53]	1.00 [.74-1.36]	.79 [.50-1.25]	.97 [.60-1.55]	.75 [.50-1.12]
**Cumulative ACEs**								
Less than 6	Ref	Ref	Ref	Ref	Ref	Ref	Ref	Ref
6 or more	1.57 [1.11-2.32]*	.98 [.53-1.80]	1.55 [1.09-2.19]*	1.33 [.95-1.86]	1.29 [.88-1.88]	.94 [.54-1.63]	.80 [.46-1.40]	1.91 [1.28-2.83]**
0	Ref	Ref	Ref	Ref	Ref	Ref	Ref	Ref
1	1.89 [.83-4.33]	1.07 [.49-2.32]	.99 [.61-1.62]	1.43 [.87-2.34]	.97 [.57-1.65]	1.09 [.51-2.33]	1.16 [.64-2.09]	1.50 [.70-3.19]
2	1.87 [.83-4.19]	1.01 [.46-2.25]	1.38 [.85-2.24]	1.23 [.77-1.98]	.98 [.57-1.68]	1.30 [.64-2.62]	1.58 [.88-2.85]	1.38 [.62-3.05]
3	2.81 [1.25-6.35]*	1.56 [.73-3.30]	1.28 [.80-2.08]	1.21 [.75-1.97]	1.54 [.90-2.61]	.97 [.45-2.12]	1.30 [.72-2.33]	2.55 [1.19-5.46]*
4+	3.75 [1.74-8.08]**	1.26 [.63-2.54]	1.47 [.94-2.31]	1.46 [.92-2.30]	1.36 [.86-2.17]	1.25 [.63-2.50]	1.18 [.62-2.25]	2.50 [1.20-5.14]*
**Clustered ACEs**								
Class 1	Ref	Ref	Ref	Ref	Ref	Ref	Ref	Ref
Class 2	1.48 [.99-2.21]	1.26 [.80-2.00]	1.26 [.93-1.71]	1.30 [.99-1.72]	1.05 [.76-1.45]	.75 [.47-1.17]	1.47 [1.06-2.05]*	1.45 [.99-2.17]
Class 3	2.58 [1.67-3.99]***	1.04 [.59-1.83]	1.18 [.83-1.68]	.91 [.64-1.28]	1.24 [.84-1.82]	1.12 [.69-1.81]	1.05 [.66-1.68]	1.61 [1.03-2.53]*
Class 4	2.56 [1.64-4.00]***	1.25 [.71-2.20]	1.56 [1.09-2.24]*	1.28 [.89-1.83]	1.18 [.79-1.73]	1.14 [.71-1.84]	1.06 [.64-1.73]	2.10 [1.35-3.27]**

*p<.05 **p<.01 ***p<.001

N/A = omitted due to within cell sizes

The table shows the fold-increase in the odds (OR, odds ratio) of each adult human capital outcome (columns) for each level of ACEs measurement (rows). Models were run separately for each of the 3 ACE measurements

Supplementary Table S5: Adjusted associations between single, cumulative and clustered prospective ACEs and health and human capital outcomes

	Psychological distress	Social isolation	Incomplete schooling	Unemployed	Substance use	HIV infection	Welfare receipt	Criminality
**Single ACEs**								
Physical abuse	1.20 [.85-1.69]	1.12 [.75-1.68]	1.69 [1.21-2.35]**	1.31 [1.02-1.69]*	1.04 [.74-1.47]	1.30 [.85-1.99]	1.33 [.90-1.99]	.86 [.528-1.29]
Sexual abuse	.95 [.65-1.37]	.64 [.42-.99]*	1.58 [1.21-2.08]**	1.20 [.92-1.57]	.87 [.62-1.22]	1.31 [.90-1.91]	1.53 [1.07-2.20]*	1.11 [.77-1.61]
Emotional abuse	1.74 [1.24-2.44]**	1.19 [.80-1.76]	1.09 [.81-1.45]	1.06 [.81-1.38]	1.37 [1.02-1.84]*	1.28 [.86-1.90]	.85 [.58-1.25]	1.36 [.95-1.93]
Child separation	.57 [.36-.91]*	1.17 [.65-2.09]	1.03 [.70-1.51]	1.05 [.74-1.49]	1.35 [.92-1.97]	1.08 [.64-1.82]	1.09 [.69-1.72]	1.21 [.72-2.01]
Parental divorce	.81 [.58-1.13]	.96 [.64-1.45]	1.22 [.90-1.65]	1.19 [.91-1.56]	1.06 [.78-1.44]	1.04 [.72-1.51]	1.31 [.89-1.92]	.78 [.55-1.12]
Parental death	1.02 [.66-1.58]	.91 [.54-1.52]	1.10 [.81-1.49]	.81 [.59-1.11]	.95 [.64-1.40]	1.26 [.82-1.93]	.87 [.57-1.32]	1.25 [.83-1.90]
Exposure to violence	1.64 [1.08-2.49]*	.95 [.56-1.62]	.87 [.61-1.25]	1.00 [.73-1.37]	1.33 [.88-2.00]	.84 [.52-1.34]	.96 [.65-1.41]	1.45 [.88-2.39]
Exposure to IPV	1.18 [.83-1.69]	1.11 [.73-1.69]	1.03 [.76-1.40]	.86 [.64-1.16]	1.39 [.95-2.02]	1.20 [.78-1.84]	.85 [.54-1.34]	2.27 [1.49-3.45]***
Household substance abuse	1.23 [.88-1.73]	1.20 [.81-1.79]	.99 [.76-1.30]	1.04 [.81-1.34]	.84 [.60-1.17]	.83 [.56-1.22]	1.01 [.70-1.46]	.84 [.59-1.21]
Chronic unemployment	.82 [.50-1.32]	1.34 [.67-2.68]	1.23 [.76-1.98]	1.10 [.75-1.63]	.83 [.52-1.34]	.69 [.38-1.27]	1.71 [.97-3.04]	.71 [.42-1.20]
Household legal trouble	1.34 [.95-1.88]	1.46 [.93-2.30]	1.17 [.87-1.59]	.95 [.74-1.23]	1.06 [.75-1.51]	1.00 [.66-1.54]	.81 [.51-1.29]	1.36 [.93-2.00]
Household illness/disability	1.04 [.74-1.47]	.71 [.47-1.06]	1.27 [.95-1.72]	1.07 [.81-1.42]	.84 [.61-1.16]	1.18 [.78-1.78]	1.33 [.83-2.14]	1.21 [.83-1.78]
Household death	1.12 [.80-1.58]	.88 [.59-1.30]	.80 [.59-1.07]	1.57 [1.17-2.11]**	1.35 [.95-1.90]	1.13 [.77-1.66]	.89 [.56-1.41]	1.15 [.82-1.61]
**Cumulative ACEs**								
Less than 6	Ref	Ref	Ref	Ref	Ref	Ref	Ref	Ref
6 or more	1.63 [1.19-2.23]**	1.08 [.75-1.57]	1.51 [1.15-1.99]**	1.28 [1.10-1.64]*	1.39 [.98-1.99]	1.36 [.95-1.95]	1.18 [.87-1.61]	1.80 [1.28-2.54]**
0	Ref	Ref	Ref	Ref	Ref	Ref	Ref	Ref
1	.47 [.09-2.43]	N/A	1.29 [.11-15.03]	1.11 [.16-7.59]	1.46 [.11-19.53]	N/A	1.49 [.09-23.76]	.34 [.02-6.37]
2	.68 [.25-1.84]	N/A	1.49 [.15-15.03]	1.28 [.21-7.64]	3.16 [.29-34.36]	N/A	1.25 [.10-16.01]	1.31 [.11-15.13]
3	.40 [.18-.88]*	1.42 [.55-3.67]	1.38 [.15-12.88]	1.48 [.27-8.21]	1.87 [.18-18.97]	.71 [.22-2.25]	1.67 [.14-19.55]	.74 [.07-7.38]
4+	N/A	N/A	2.31 [.26-20.57]	1.72 [.33-8.98]	2.71 [.29-25.48]	N/A	2.19 [.21-23.13]	1.22 [.14-10.88]
**Clustered ACEs**								
Class 1	Ref	Ref	Ref	Ref	Ref	Ref	Ref	Ref
Class 2	1.40 [.65-3.00]	1.08 [.46-2.55]	1.77 [.97-3.20]	1.33 [.79-2.24]	.98 [.56-1.72]	1.01 [.46-2.19]	1.67 [.87-3.18]	1.21 [.52-2.84]
Class 3	2.23 [1.10-5.04]*	1.41 [.55-3.52]	1.51 [.81-2.81]	1.08 [.62-1.86]	1.46 [.78-2.73]	1.24 [.54-2.89]	1.36 [.63-2.91]	2.29 [1.01-5.22]*
Class 4	2.47 [1.19-5.09]*	1.25 [.55-2.86]	2.39 [1.33-4.31]**	1.56 [.93-2.67]	1.58 [.84-2.98]	1.33 [.62-2.86]	1.96 [.97-3.93]	2.60 [1.12-6.02]*

$^{*}p<.05$ $^{**}p<.01$ $^{***}p<.001$
N/A = omitted due to within cell sizes
The table shows the fold-increase in the odds (OR, odds ratio) of each adult human capital outcome (columns) for each level of ACEs measurement (rows). Models were run separately for each of the 3 ACE measurements and fully adjusted for all covariates, including sex, SES at birth, SES at age 12, SES at age 22, maternal age at birth and maternal and paternal schooling.

Supplementary Table S6: Adjusted associations between single, cumulative and clustered retrospective ACEs and health and human capital outcomes

	Psychological distress	Social isolation	Incomplete schooling	Unemployed	Substance use	HIV infection	Welfare receipt	Criminality
**Single ACEs**								
Physical abuse	1.32 [.76-2.28]	1.92 [.93-3.98]	1.16 [.66-2.04]	1.02 [.63-1.64]	1.62 [.89-2.96]	1.03 [.51-2.08]	.79 [.38-1.67]	1.37 [.72-2.59]
Sexual abuse	2.05 [1.02-4.12]*	1.83 [.75-4.50]	1.15 [.53-2.48]	.80 [.39-1.66]	1.50 [.66-3.40]	3.03 [1.03-7.91]*	1.09 [.43-2.72]	1.90 [.80-4.50]
Emotional abuse	1.96 [1.37-2.82]***	.82 [.53-1.27]	1.48 [1.09-2.01]*	1.04 [.79-1.36]	1.02 [.71-1.48]	1.30 [.80-2.11]	1.10 [.77-1.57]	1.35 [.94-1.92]
Parental divorce	.82 [.59-1.13]	.64 [.41-.99]*	.81 [.61-1.07]	1.04 [.75-1.46]	.96 [.66-1.40]	.85 [.47-1.51]	1.12 [.71-1.78]	.95 [.63-1.43]
Parental death	1.17 [.80-1.72]	1.39 [.85-2.26]	1.17 [.84-1.62]	.89 [.64-1.22]	.79 [.53-1.17]	1.09 [.73-1.64]	.79 [.50-1.22]	1.30 [.88-1.92]
Exposure to violence	1.05 [.71-1.55]	1.27 [.80-2.01]	.97 [.72-1.30]	.95 [.71-1.26]	1.73 [1.28-2.35]***	.97 [.60-1.55]	.92 [.64-1.33]	1.47 [.97-2.22]
Exposure to IPV	.95 [.60-1.53]	.57 [.28-1.13]	.92 [.61-1.37]	.69 [.45-1.04]	1.05 [.64-1.72]	.72 [.39-1.32]	.81 [.49-1.34]	1.32 [.80-2.18]
Household substance abuse	1.39 [.96-2.02]	1.61 [1.04-2.50]*	1.03 [.72-1.48]	1.56 [1.19-2.06]**	1.16 [.81-1.65]	1.50 [1.00-2.24]*	.92 [.59-1.43]	1.07 [.74-1.57]
Chronic unemployment	1.14 [.81-1.61]	1.26 [.81-1.97]	.92 [.67-1.25]	1.10 [.85-1.42]	.99 [.73-1.34]	.68 [.42-1.09]	1.27 [.92-1.76]	1.29 [.90-1.87]
Household legal trouble	.88 [.60-1.31]	.71 [.42-1.21]	.94 [.68-1.29]	.92 [.65-1.31]	1.12 [.75-1.69]	1.19 [.75-1.87]	.98 [.62-1.53]	1.06 [.73-1.55]
Household illness/disability	1.71[1.16-2.48]**	.86 [.55-1.36]	.93 [.68-1.27]	.76 [.58-.99]*	.77 [.57-1.06]	.70 [.45-1.09]	.99 [.67-1.46]	.87 [.52-1.44]
Household death	.91 [.64-1.30]	1.04 [.565-1.36]	.87 [.63-1.19]	1.20 [.90-1.60]	.97 [.70-1.34]	.79 [.49-1.26]	.95 [.55-1.65]	.71 [.46-1.08]
**Cumulative ACEs**								
Less than 6	Ref	Ref	Ref	Ref	Ref	Ref	Ref	Ref
6 or more	1.72 [1.13-2.61]*	.88 [.46-1.69]	1.21 [.83-1.76]	1.12 [.78-1.59]	1.22 [.82-1.82]	.84 [.47-1.49]	.83 [.44-1.57]	1.69 [1.09-2.63]*
0	Ref	Ref	Ref	Ref	Ref	Ref	Ref	Ref
1	1.88 [.82-4.30]	1.06 [.47-2.39]	.76 [.45-1.28]	1.29 [.76-2.00]	.92 [.53-1.59]	.97 [.44-2.13]	1.14 [.58-2.22]	1.55 [.69-3.48]
2	1.71 [.76-3.84]	1.08 [.47-2.49]	1.14 [.68-1.91]	1.07 [.65-1.77]	1.07 [.61-1.88]	1.12 [.54-2.30]	1.35 [.70-2.60]	1.60 [.69-3.70]
3	2.87 [1.26-6.54]**	1.49 [.67-3.29]	.93 [.56-1.55]	1.01 [.60-1.68]	1.48 [.84-2.61]	.79 [.35-1.80]	1.27 [.65-2.51]	2.54 [1.13-5.72]*
4+	4.08 [1.88-6.85]***	1.15 [.54-2.44]	.93 [.57-1.51]	1.09 [.67-1.78]	1.31 [.80-2.16]	1.02 [.49-2.10]	1.17 [.55-2.46]	2.43 [1.08-5.46]*
**Clustered ACEs**								
Class 1	Ref	Ref	Ref	Ref	Ref	Ref	Ref	Ref
Class 2	1.52 [1.01-2.29]*	1.18 [.73-1.92]	.95 [.68-1.32]	1.05 [.78-1.40]	1.08 [.76-1.53]	.62 [.37-1.03]	1.41 [.98-2.05]	1.51 [.97-2.37]
Class 3	2.82 [1.8 -4.41] ***	1.01 [.57-1.81]	1.05 [.72-1.52]	.80 [.57-1.14]	1.27 [.84-1.93]	1.07 [.65-1.77]	1.01 [.60-1.70]	1.69 [1.04-2.73]*
Class 4	2.97 [1.86-4.74]***	1.09 [.58-2.04]	.98 [.66-1.46]	.94 [.63-1.39]	1.15 [.74-1.78]	.88 [.50-1.53]	1.08 [.61-1.92]	2.01 [1.18-3.29]**

* $p<.05$ ** $p<.01$ *** $p<.001$

N/A = omitted due to within cell sizes

The table shows the fold-increase in the odds (OR, odds ratio) of each adult human capital outcome (columns) for each level of ACEs measurement (rows). Models were run separately for each of the 3 ACE measurements and fully adjusted for all covariates, including sex, SES at birth, SES at age 12, SES at age 22, maternal age at birth and maternal and paternal schooling.

Supplementary Table S7: Adjusted associations between single, cumulative and clustered prospective ACEs and health and human capital outcomes, by sex

	Psychological distress		Social isolation		Incomplete schooling		Unemployed		Substance use		HIV infection		Welfare receipt		Criminality	
	Male	Female	Male	Female	Male	Female	Male	Female	Male	Female	Male	Female	Male	Female	Male	Female
**Single ACEs**																
Physical abuse	1.16 [0.64-2.12]	1.29 [0.86-1.95]	0.8 [0.48-1.35]	1.8 [0.88-3.66]	1.46 [0.96-2.21]	2.08*** [1.25-3.45]	1.28 [0.87-1.89]	1.37 [0.95-1.97]	1.03 [0.66-1.6]	1.05 [0.6-1.83]	1.47 [0.77-2.82]	1.22 [0.71-2.09]	1.3 [0.6-2.82]	1.36 [0.92-2.02]	0.89 [0.57-1.38]	0.79 [0.35-1.78]
Sexual abuse	0.93 [0.47-1.82]	0.96 [0.62-1.48]	0.75 [0.44-1.29]	0.53 [0.25-1.09]	1.68*** [1.17-2.4]	1.52* [1.01-2.31]	1.41 [0.95-2.09]	1.08 [0.72-1.6]	0.75 [0.51-1.1]	1.07 [0.59-1.93]	1.49 [0.76-2.95]	1.2 [0.72-2.00]	1.51 [0.74-3.11]	1.56* [1.06-2.3]	0.97 [0.62-1.5]	1.86 [0.91-3.79]
Emotional abuse	2.54** [1.42-4.53]	1.44 [0.93-2.22]	1.03 [0.59-1.81]	1.47 [0.7-3.09]	1.12 [0.75-1.66]	1.05 [0.65-1.71]	1.04 [0.7-1.55]	1.06 [0.74-1.52]	1.52* [1.03-2.25]	1.20 [0.71-2.05]	1.70 [0.96-2.99]	1.08 [0.65-1.8]	0.66 [0.28-1.56]	0.94 [0.63-1.41]	1.57* [1.04-2.38]	0.94 [0.39-2.22]
Child separation	0.51 [0.21-1.24]	0.6 [0.34-1.06]	1.46 [0.68-3.15]	0.81 [0.29-2.29]	0.88 [0.49-1.6]	1.2 [0.69-2.06]	1.06 [0.6-1.86]	1.04 [0.64-1.69]	1.13 [0.64-1.97]	1.6 [0.93-2.74]	0.72 [0.27-1.93]	1.25 [0.68-2.31]	1.26 [0.48-3.28]	1.02 [0.63-1.66]	1.29 [0.74-2.24]	1.21 [0.45-3.24]
Parental divorce	0.72 [0.36-1.41]	0.82 [0.53-1.24]	0.83 [0.48-1.42]	1.03 [0.54-1.95]	1.28 [0.82-2.02]	1.13 [0.75-1.71]	1.15 [0.79-1.68]	1.23 [0.85-1.78]	0.93 [0.65-1.35]	1.30 [0.75-2.23]	0.79 [0.41-1.54]	1.28 [0.81-2.03]	1.44 [0.7-2.93]	1.29 [0.84-1.97]	0.74 [0.48-1.12]	1.03 [0.46-2.32]
Parental death	0.86 [0.39-1.89]	1.14 [0.69-1.88]	0.73 [0.36-1.5]	1.43 [0.66-3.07]	0.85 [0.55-1.31]	1.63 [0.97-2.72]	0.82 [0.52-1.28]	0.79 [0.51-1.22]	0.92 [0.57-1.47]	1.01 [0.53-1.95]	1.4 [0.75-2.65]	1.09 [0.57-2.09]	0.71 [0.24-2.14]	0.96 [0.62-1.5]	1.06 [0.64-1.76]	1.92 [0.77-4.79]
Exposure to violence	3.05* [1.01-9.18]	1.52 [0.97-2.39]	1.34 [0.62-2.89]	0.68 [0.33-1.41]	0.94 [0.54-1.64]	0.81 [0.52-1.27]	1.03 [0.58-1.81]	1.01 [0.69-1.49]	1.57 [0.9-2.77]	1.04 [0.62-1.77]	0.88 [0.36-2.2]	0.80 [0.46-1.41]	0.69 [0.25-1.87]	1.05 [0.69-1.61]	1.60 [0.85-3.02]	1.46 [0.62-3.46]
Exposure to IPV	1.18 [0.57-2.44]	1.05 [0.68-1.64]	0.83 [0.47-1.49]	1.6 [0.77-3.3]	1.18 [0.79-1.79]	0.81 [0.51-1.3]	0.79 [0.53-1.18]	0.95 [0.64-1.42]	1.42 [0.89-2.25]	1.26 [0.64-2.49]	0.92 [0.47-1.83]	1.43 [0.86-2.37]	0.94 [0.37-2.4]	0.86 [0.51-1.45]	2.16** [1.31-3.55]	2.02 [0.81-5.06]
Household substance abuse	1.28 [0.68-2.42]	1.21 [0.81-1.82]	1.71 [0.97-3.02]	0.78 [0.41-1.5]	1.00 [0.68-1.47]	1.01 [0.65-1.58]	1.12 [0.77-1.64]	0.99 [0.67-1.45]	0.79 [0.5-1.26]	0.88 [0.54-1.43]	0.62 [0.31-1.25]	1.00 [0.61-1.63]	0.89 [0.38-2.12]	1.05 [0.72-1.54]	0.85 [0.55-1.32]	0.83 [0.38-1.79]
Chronic unemployment	0.52 [0.21-1.26]	0.98 [0.54-1.77]	0.88 [0.36-2.13]	2.37 [0.7-8.07]	0.89 [0.46-1.7]	1.87 [0.81-4.32]	0.78 [0.41-1.49]	1.49 [0.89-2.52]	1.01 [0.55-1.86]	0.6 [0.29-1.25]	0.75 [0.3-1.91]	0.56 [0.27-1.16]	1.26 [0.39-4.02]	2.03* [1.07-3.86]	0.77 [0.38-1.59]	0.6 [0.22-1.61]
Household legal trouble	1.72 [0.91-3.26]	1.15 [0.74-1.78]	1.63 [0.94-2.83]	1.39 [0.65-2.97]	1.46 [0.96-2.21]	0.91 [0.56-1.47]	0.97 [0.67-1.41]	0.94 [0.65-1.37]	1.15 [0.77-1.72]	0.94 [0.53-1.66]	1.03 [0.56-1.91]	1.03 [0.58-1.82]	0.79 [0.27-2.28]	0.81 [0.54-1.23]	1.46 [0.97-2.2]	1.21 [0.53-2.76]
Household illness/disability	1.46 [0.77-2.77]	0.89 [0.57-1.4]	0.91 [0.53-1.58]	0.56 [0.27-1.15]	1.68 [1.17-2.4]	1.33 [0.85-2.07]	1.34 [0.91-1.96]	0.87 [0.57-1.34]	0.72 [0.48-1.08]	1.11 [0.61-2.04]	1.49 [0.79-2.8]	1.02 [0.59-1.76]	1.62 [0.68-3.87]	1.24 [0.76-2.05]	1.26 [0.81-1.94]	0.99 [0.47-2.12]
Household death	1.3 [0.68-2.46]	0.97 [0.64-1.47]	0.93 [0.56-1.56]	0.79 [0.41-1.51]	1.12 [0.75-1.66]	0.67 [0.42-1.06]	1.55* [1.03-2.33]	1.65* [1.09-2.5]	1.31 [0.85-2.03]	1.39 [0.8-2.4]	1.14 [0.62-2.09]	1.13 [0.67-1.89]	0.96 [0.42-2.22]	0.87 [0.56-1.36]	1.27 [0.82-1.96]	0.95 [0.39-2.34]
**Cumulative ACEs**																
Less than 6	Ref	Ref	Ref	Ref	Ref	Ref	Ref	Ref	Ref	Ref	Ref	Ref	Ref	Ref	Ref	Ref
6 or more	1.97* [1.11-3.48]	1.44 [0.98-2.11]	1.26 [0.76-2.07]	0.88 [0.46-1.67]	1.74** [1.2-2.53]	1.24 [0.8-1.91]	1.47* [1.04-2.09]	1.16 [0.8-1.69]	1.34 [0.91-1.98]	1.47 [0.79-2.71]	1.43 [0.82-2.48]	1.37 [0.81-2.32]	0.94 [0.39-2.24]	1.29 [0.92-1.82]	1.84** [1.22-2.76]	1.8 [0.93-3.49]
0	Ref	Ref	Ref	Ref	Ref	Ref	Ref	Ref	Ref	Ref	Ref	Ref	Ref	Ref	Ref	Ref
1	N/A	0.65 [0.11-3.83]	N/A	N/A	0.39 [0.02-7.99]	0.59 [0.08-4.4]	0.11 [0.01-2.41]	0.8 [0.2-3.26]	1.03 [0.06-19.19]	N/A	N/A	N/A	N/A	1.3 [0.08-22.15]	0.21 [0.01-5.1]	N/A
2	N/A	0.88 [0.3-2.58]	N/A	N/A	0.66 [0.04-11.22]	0.57 [0.15-2.19]	0.34 [0.02-6.4]	0.51 [0.18-1.49]	2.02 [0.12-33.19]	N/A	N/A	N/A	N/A	1.06 [0.07-15.6]	0.87 [0.06-13.82]	N/A
3	N/A	0.47 [0.18-1.18]	N/A	N/A	0.39 [0.03-5.37]	0.75 [0.32-1.74]	0.15 [0.01-2.22]	1.04 [0.57-1.93]	1.17 [0.09-16.16]	0.54 [0.06-4.5]	0.82 [0.13-4.97]	0.65 [.13-3.33]	N/A	1.48 [0.12-17.87]	0.36 [0.03-4.89]	1.09 [0.16-7.48]
4+	N/A	N/A	N/A	N/A	0.77 [0.06-9.67]	N/A	0.21 [0.02-2.89]	N/A	1.77 [0.14-21.9]	N/A	N/A	N/A	N/A	2.06 [0.17-24.36]	0.67 [0.06-7.9]	N/A
**LCA derived clusters**																
Class 2	0.29 [0.08-1.05]	1.14 [0.52-2.47]	1.24 [0.34-4.46]	0.9 [0.27-3.04]	1.77 [0.67-4.65]	1.67 [0.76-3.69]	0.92 [0.39-2.16]	1.74 [0.91-3.36]	0.86 [0.39-1.89]	1.16 [0.48-2.82]	1.42 [0.37-5.41]	0.79 [0.32-1.96]	N/A	1.61 [0.85-3.02]	1.25 [0.47-3.32]	1.37 [0.31-6.04]
Class 3	0.74 [0.24-2.31]	1.53 [0.59-4]	1.16 [0.3-4.42]	1.77 [0.49-6.33]	1.67 [0.61-4.59]	1.25 [0.48-3.26]	0.62 [0.26-1.5]	1.74 [0.82-3.7]	1.58 [0.72-3.46]	1.23 [0.42-3.55]	1.13 [0.25-5.12]	1.34 [0.52-3.48]	N/A	1.4 [0.68-2.89]	2.07 [0.77-5.57]	2.98 [0.67-13.24]
Class 4	N/A	1.64 [0.74-3.65]	1.57 [0.45-5.46]	0.81 [0.24-2.76]	2.5 [0.98-6.42]	2.09 [0.87-5.02]	1.19 [0.51-2.8]	1.9 [0.97-3.74]	1.56 [0.74-3.31]	1.62 [0.58-4.52]	1.59 [0.41-6.12]	1.12 [0.45-2.78]	N/A	2.11* [1.1-4.05]	2.84 [1.1-7.34]*	2.21 [0.44-11.12]

* p<.05 ** p<.01 *** p<.001; N/A = omitted due to within cell sizes

The table shows the fold-increase in the odds (OR, odds ratio) of each adult human capital outcome (columns) for each level of ACEs measurement (rows). Models were run separately for each of the 3 ACE measurements and fully adjusted for all covariates, including sex, SES at birth, SES at age 12, SES at age 22, maternal age at birth and maternal and paternal schooling.

Supplementary Table S8: Adjusted associations between single, cumulative and clustered retrospective ACEs and health and human capital outcomes, by sex

	Psychological distress		Social isolation		Incomplete schooling		Unemployed		Substance use		HIV infection		Welfare receipt		Criminality	
	Male	Female	Male	Female	Male	Female	Male	Female	Male	Female	Male	Female	Male	Female	Male	Female
**Single ACEs**																
Physical abuse	1.39 [0.6-3.22]	1.19 [0.53-2.69]	0.96 [0.33-2.74]	6.02** [1.93-8.85]	0.91 [0.45-1.85]	1.73 [0.66-4.52]	0.98 [0.51-1.87]	1.27 [0.56-2.85]	1.74 [0.85-3.56]	1.4 [0.32-6.09]	1.26 [0.49-3.26]	0.80 [0.26-2.44]	0.65 [0.12-3.4]	0.94 [0.42-2.11]	1.44 [0.71-2.91]	1.07 [0.21-5.49]
Sexual abuse	1.61 [0.35-7.39]	2.20 [0.97-5.01]	1.97 [0.43-8.98]	1.59 [0.42-5.97]	1.6 [0.49-5.24]	0.91 [0.29-2.79]	0.66 [0.2-2.21]	0.9 [0.36-2.24]	1.24 [0.38-4.09]	1.69 [0.59-4.82]	2.8 [0.27-29.35]	3.32* [1.01-10.91]	1.48 [0.18-12]	0.93 [0.37-2.37]	1.82 [0.56-5.93]	3.25 [0.9-11.76]
Emotional abuse	1.88 [0.93-3.79]	2.15** [1.39-3.32]	0.74 [0.4-1.36]	0.93 [0.44-1.96]	1.47 [0.94-2.3]	1.6* [1.02-2.49]	1.17 [0.8-1.73]	0.93 [0.62-1.39]	1.23 [0.77-1.95]	0.78 [0.44-1.39]	1.33 [0.67-2.68]	1.21 [0.68-2.15]	1.13 [0.59-2.16]	1.09 [0.71-1.67]	1.38 [0.91-2.1]	1.46 [0.66-3.24]
Parental divorce	0.83 [0.47-1.49]	0.79 [0.52-1.21]	0.52* [0.28-1]	0.78 [0.38-1.6]	0.88 [0.57-1.36]	0.7 [0.45-1.1]	0.9 [0.6-1.35]	1.21 [0.76-1.92]	0.86 [0.54-1.36]	1.10 [0.63-1.93]	0.84 [0.4-1.77]	0.87 [0.47-1.62]	1.02 [0.42-2.49]	1.18 [0.77-1.81]	0.84 [0.52-1.36]	1.49 [0.6-3.68]
Parental death	0.97 [0.5-1.88]	1.36 [0.84-2.20]	1.26 [0.68-2.31]	1.54 [0.68-3.46]	1.1 [0.7-1.74]	1.26 [0.79-1.99]	1.05 [0.69-1.59]	0.7 [0.45-1.11]	0.69 [0.45-1.06]	0.93 [0.45-1.94]	1.21 [0.67-2.21]	1.02 [0.59-1.77]	0.82 [0.32-2.09]	0.78 [0.49-1.25]	1.03 [0.66-1.62]	2.21 [0.93-5.26]
Exposure to violence	0.92 [0.49-1.72]	1.17 [0.72-1.92]	1.15 [0.64-2.06]	1.51 [0.71-3.2]	0.92 [0.62-1.38]	1.02 [0.62-1.69]	0.94 [0.63-1.42]	1.00 [0.67-1.49]	1.96** [1.3-2.96]	1.52 [0.85-2.75]	0.90 [0.44-1.83]	1.04 [0.57-1.89]	1.02 [0.45-2.33]	0.86 [0.54-1.37]	1.58 [0.99-2.53]	1.11 [0.42-2.95]
Exposure to IPV	0.91 [0.36-2.29]	1.10 [0.59-2.04]	0.74 [0.3-1.82]	0.24 [0.05-1.15]	1.03 [0.6-1.76]	0.78 [0.39-1.57]	0.97 [0.56-1.66]	0.46* [0.23-0.94]	0.87 [0.46-1.62]	1.48 [0.64-3.43]	0.77 [0.29-2.03]	0.69 [0.32-1.49]	0.95 [0.22-4.1]	0.72 [0.4-1.31]	1.44 [0.77-2.68]	1.01 [0.3-3.36]
Household substance abuse	2.08* [1.09-3.99]	1.01 [0.62-1.63]	1.56 [0.86-2.84]	1.87 [0.91-3.85]	1.17 [0.71-1.91]	0.9 [0.54-1.48]	1.8** [1.21-2.66]	1.38 [0.85-2.24]	1.31 [0.85-2.03]	0.82 [0.42-1.62]	1.37 [0.72-2.6]	1.68 [0.98-2.87]	0.96 [0.43-2.18]	0.9 [0.54-1.48]	1.25 [0.82-1.91]	0.72 [0.26-2.04]
Chronic unemployment	1 [0.56-1.79]	1.27 [0.83-1.95]	1.7 [0.96-3.02]	0.87 [0.42-1.78]	0.85 [0.55-1.31]	1.05 [0.68-1.61]	1.16 [0.75-1.77]	1.02 [0.71-1.47]	0.96 [0.64-1.43]	0.97 [0.58-1.64]	0.65 [0.31-1.34]	0.67 [0.37-1.2]	0.95 [0.42-2.13]	1.47* [1.02-2.11]	1.38 [0.9-2.11]	1.08 [0.47-2.47]
Household legal trouble	0.85 [0.42-1.7]	0.94 [0.57-1.56]	0.61 [0.3-1.24]	0.97 [0.39-2.41]	1.04 [0.68-1.59]	0.87 [0.52-1.46]	0.9 [0.57-1.42]	0.96 [0.57-1.6]	0.98 [0.61-1.57]	1.49 [0.77-2.86]	1.54 [0.82-2.88]	0.88 [0.44-1.76]	0.61 [0.28-1.33]	1.17 [0.63-2.16]	1.09 [0.71-1.68]	0.99 [0.41-2.41]
Household illness/disability	1.53 [0.77-3.04]	1.88** [1.18-2.98]	0.74 [0.4-1.37]	1.07 [0.53-2.19]	0.97 [0.64-1.48]	0.88 [0.54-1.43]	0.71 [0.46-1.1]	0.78 [0.54-1.11]	0.92 [0.59-1.45]	0.58 [0.34-1.00]	0.88 [0.47-1.66]	0.57* [0.33-0.99]	1.07 [0.47-2.45]	0.95 [0.63-1.44]	0.74 [0.44-1.25]	1.44 [0.58-3.55]
Household death	0.83 [0.44-1.55]	0.91 [0.59-1.42]	1.01 [0.57-1.78]	1.03 [0.54-1.99]	0.78 [0.47-1.3]	0.95 [0.61-1.48]	1.25 [0.84-1.88]	1.18 [0.8-1.75]	1.06 [0.72-1.54]	0.84 [0.47-1.53]	0.96 [0.43-2.14]	0.69 [0.39-1.21]	0.89 [0.29-2.76]	0.97 [0.61-1.53]	0.74 [0.45-1.22]	0.64 [0.27-1.49]
**Cumulative ACEs**																
Less than 6	Ref	Ref	Ref	Ref	Ref	Ref	Ref	Ref	Ref	Ref	Ref	Ref	Ref	Ref	Ref	Ref
6 ore more	1.46 [0.76-2.82]	2.04 [1.17-3.58]	0.7 [0.29-1.7]	1.26 [0.51-3.08]	1.27 [0.79-2.03]	1.15 [0.62-2.14]	1.43 [0.84-2.42]	0.85 [0.5-1.45]	1.34 [0.8-2.24]	1.02 [0.48-2.18]	1.09 [0.48-2.46]	0.66 [0.3-1.46]	0.54 [0.16-1.86]	1.03 [0.54-1.98]	1.47 [0.87-2.48]	2.77* [1.18-6.5]
0	Ref	Ref	Ref	Ref	Ref	Ref	Ref	Ref	Ref	Ref	Ref	Ref	Ref	Ref	Ref	Ref
1	1.2 [0.3-4.88]	2.60 [0.93-7.31]	0.66 [0.25-1.77]	2.67 [0.44-16.12]	0.74 [0.35-1.57]	0.79 [0.35-1.74]	0.87 [0.39-1.91]	1.75 [0.91-3.39]	0.97 [0.45-2.09]	0.84 [0.35-2]	0.7 [0.2-2.47]	1.25 [0.44-3.53]	1.44 [0.32-6.52]	1.09 [0.51-2.34]	2.31 [0.8-6.73]	0.74 [0.2-2.74]
2	1.39 [0.31-6.18]	2.06 [0.73-5.82]	0.91 [0.34-2.43]	1.85 [0.31-10.91]	1.04 [0.49-2.2]	1.27 [0.6-2.68]	1.05 [0.47-2.35]	1.08 [0.57-2.07]	1.29 [0.62-2.68]	0.86 [0.36-2.05]	0.9 [0.25-3.22]	1.36 [0.53-3.49]	1.25 [0.21-7.52]	1.44 [0.72-2.87]	3.10* [1.05-9.14]	0.42 [0.1-1.8]
3	2.07 [0.56-7.66]	3.43* [1.13-7.38]	0.91 [0.35-2.4]	3.76 [0.59-23.96]	0.67 [0.31-1.45]	1.34 [0.62-2.89]	1.18 [0.56-2.49]	0.85 [0.42-1.71]	1.96 [0.89-4.31]	1.05 [0.44-2.51]	0.7 [0.19-2.57]	0.88 [0.28-2.74]	1.42 [0.24-8.3]	1.26 [0.62-2.55]	3.97** [1.41-11.21]	1.06 [0.29-3.91]
4+	2.87 [0.8-10.29]	5.44*** [2.04-6.51]	0.70 [0.28-1.73]	2.92 [0.51-16.55]	0.95 [0.48-1.88]	0.9 [0.44-1.86]	1.17 [0.56-2.42]	1.01 [0.52-1.95]	1.59 [0.82-3.09]	1.04 [0.47-2.3]	1.05 [0.33-3.3]	1.02 [0.39-2.67]	1.14 [0.18-7.32]	1.24 [0.59-2.58]	3.64** [1.27-10.47]	1.18 [0.36-3.82]
**Clustered ACEs**																
Class 2	1.45 [0.71-2.99]	1.55 [0.93-2.57]	1.26 [0.69-2.31]	1.04 [0.46-2.34]	0.9 [0.56-1.46]	0.97 [0.61-1.53]	1.11 [0.69-1.8]	1.00 [0.66-1.53]	0.93 [0.59-1.47]	1.33 [0.79-2.25]	0.53 [0.22-1.25]	0.65 [0.35-1.24]	1.09 [0.42-2.83]	1.60* [1.06-2.41]	1.88* [1.12-3.14]	0.69 [0.25-1.85]
Class 3	2.8* [1.18-6.64]	2.71*** [1.58-4.63]	0.6 [0.22-1.69]	1.63 [0.7-3.82]	1.17 [0.71-1.92]	0.91 [0.5-1.65]	0.97 [0.58-1.63]	0.7 [0.41-1.2]	1.16 [0.68-1.98]	1.48 [0.8-2.74]	1.13 [0.51-2.49]	1.03 [0.55-1.95]	1.11 [0.36-3.41]	0.95 [0.55-1.67]	2.12** [1.2-3.74]	0.95 [0.37-2.43]
Class 4	2.50* [1.16-5.37]	3.83*** [2.08-7.05]	1.30 [0.61-2.77]	0.69 [0.23-2.05]	1.02 [0.6-1.72]	1.01 [0.55-1.88]	1.35 [0.74-2.47]	0.61 [0.33-1.13]	1.31 [0.78-2.2]	0.74 [0.24-2.31]	1.01 [0.39-2.61]	0.75 [0.37-1.5]	0.75 [0.24-2.33]	1.30 [0.7-2.43]	2.01* [1.07-3.79]	1.99 [0.8-4.97]

$^{*}p<.05$ $^{**}p<.01$ $^{***}p<.001$; N/A = omitted due to within cell sizes

The table shows the fold-increase in the odds (OR, odds ratio) of each adult human capital outcome (columns) for each level of ACEs measurement (rows). Models were run separately for each of the 3 ACE measurements and fully adjusted for all covariates, including sex, SES at birth, SES at age 12, SES at age 22, maternal age at birth and maternal and paternal schooling.
